# RUNX1 interacts with lncRNA SMANTIS to regulate monocytic cell functions

**DOI:** 10.1038/s42003-024-06794-2

**Published:** 2024-09-13

**Authors:** Lisa M. Weiss, Timothy Warwick, Simonida Zehr, Stefan Günther, Sebastian Wolf, Tessa Schmachtel, Judit Izquierdo Ponce, Katalin Pálfi, Tom Teichmann, Alicia Schneider, Julia Stötzel, Stefan Knapp, Andreas Weigert, Rajkumar Savai, Michael A. Rieger, Thomas Oellerich, Ilka Wittig, James A. Oo, Ralf P. Brandes, Matthias S. Leisegang

**Affiliations:** 1 Goethe University Frankfurt, Institute for Cardiovascular Physiology, Frankfurt, Germany; 2 German Center of Cardiovascular Research (DZHK), Partner site RheinMain, Frankfurt, Germany; 3 Max Planck Institute for Heart and Lung Research, Bad Nauheim, Germany; 4 Goethe University Frankfurt, University Hospital, Department of Medicine II, Hematology/Oncology, Frankfurt, Germany; 5 Goethe University Frankfurt, Institute for Pharmaceutical Chemistry, Buchman Institute for Life Sciences Campus Riedberg, Frankfurt, Germany; 6 Goethe University Frankfurt, Institute of Biochemistry I, Frankfurt, Germany; 7 Lung Microenvironmental Niche in Cancerogenesis, Institute for Lung Health (ILH), Justus Liebig University Giessen, Giessen, Germany; 8 German Cancer Consortium (DKTK), Partner Site Frankfurt/Mainz, Frankfurt, Germany; 9 University Cancer Center (UCT) Frankfurt, University Hospital, Goethe University, Frankfurt, Germany; 10 Frankfurt Cancer Institute, Goethe University, Frankfurt, Germany; 11 Goethe University Frankfurt, Functional Proteomics, Institute for Cardiovascular Physiology, Frankfurt, Germany

**Keywords:** Long non-coding RNAs, Transcription

## Abstract

Monocytes, the circulating macrophage precursors, contribute to diseases like atherosclerosis and asthma. Long non-coding RNAs (lncRNAs) have been shown to modulate the phenotype and inflammatory capacity of monocytes. We previously discovered the lncRNA *SMANTIS*, which contributes to cellular phenotype expression by controlling BRG1 in mesenchymal cells. Here, we report that *SMANTIS* is particularly highly expressed in monocytes and lost during differentiation into macrophages. Moreover, different types of myeloid leukemia presented specific *SMANTIS* expression patterns. Interaction studies revealed that *SMANTIS* binds RUNX1, a transcription factor frequently mutated in AML, primarily through its Alu-element on the RUNT domain. RNA-seq after CRISPR/Cas9-mediated deletion of *SMANTIS* or RUNX1 revealed an association with cell adhesion and both limited the monocyte adhesion to endothelial cells. Mechanistically, *SMANTIS* KO reduced RUNX1 genomic binding and altered the interaction of RUNX1 with EP300 and CBFB. Collectively, *SMANTIS* interacts with RUNX1 and attenuates monocyte adhesion, which might limit monocyte vascular egress.

## Abstract (web summary)

The long non-coding RNA SMANTIS interacts with the transcription factor RUNX1 in an Alu-RUNT-dependent manner in monocytes and limits monocyte adhesion to endothelial cells.

## Introduction

Long non-coding RNAs (lncRNAs) are transcripts longer than 200 nucleotides sharing mRNA-like structural features, but lacking apparent protein-coding potential^1^. Compared to mRNAs, lncRNAs are typically less well conserved and often exhibit a cell type- and tissue-specific expression pattern^1–4^. Functionally, lncRNAs have diverse roles in biology, but many of them regulate gene expression by recruiting or inhibiting epigenetic modifiers^1,3^.

LncRNA *SMANTIS* (SMARCA4 Interacting SWI/SNF Chromatin Remodeling Complex Scaffold LncRNA), formerly known as *MANTIS*, was initially observed in endothelial cells^5^. *SMANTIS* maintains angiogenic gene expression of endothelial cells by recruiting the chromatin-remodeling SWI/Sucrose Non-Fermentable (SWI/SNF) complex, specifically Brahma-related gene-1 (BRG1) to certain sites in the genome^5^. Furthermore, *SMANTIS* prevents BRG1 binding to the Intercellular Adhesion Molecule 1 (*ICAM1*) promoter, leading to limited ICAM1-mediated adhesion of endothelial cells to monocytes^6^. Interactions of monocytes and endothelial cells are crucial for tissue remodelling, transmigration, and inflammatory responses under physiological and pathophysiological conditions^7^.

Only a few studies exist that focus on individual lncRNAs in bone-marrow-derived cells, such as macrophages and monocytes. For example, lncRNA *Morrbid* is required for the survival of neutrophils, eosinophils, and monocytes because it maintains an epigenetic repressive signature *in cis* on the promoter of the pro-apoptotic *Bcl2l11*^8^. LncRNA *DRAIR* is downregulated in monocytes from patients with type 2 diabetes and is important for the expression of anti-inflammatory and macrophage differentiation genes^9^. *PIRAT* and *LUCAT1* maintain the activity of the lineage committing transcription factors PU.1 and STAT1^10^. *RUNXOR*, a runt-related transcription factor 1 (*RUNX1*)-overlapping RNA, is upregulated in the bone marrow of patients suffering from acute myeloid leukemia (AML)^11^. LncRNA *LOUP*, which is required for myeloid differentiation and inhibition of cell growth, recruits RUNX1 to the enhancer and the promoter of *PU.1*^12^. Probably through the interaction with *PU.1*, RUNX1 is in fact essential for hematopoiesis^13^ and interestingly, this transcription factor is also frequently mutated in AML^14^. In certain types of AML, RUNX1 is fused to ETO, which decreases chromatin accessibility at the *LOUP* locus and ultimately leads to attenuated *PU.1* expression^12^.

Screening in different cell types revealed elevated expression of *SMANTIS* in peripheral blood mononuclear cells (PBMCs). Among the subpopulations of PBMCs, monocytes exhibited the highest expression levels compared to lymphocytes and granulocytes. Consequently, our goal was to elucidate the role of *SMANTIS* in monocytic cells. Our findings revealed that *SMANTIS* plays a crucial role in monocytic cell functions through its interaction with RUNX1.

## Results

### *SMANTIS* is highly expressed in monocytes and altered in acute myeloid leukemia

**Fig. 1:**
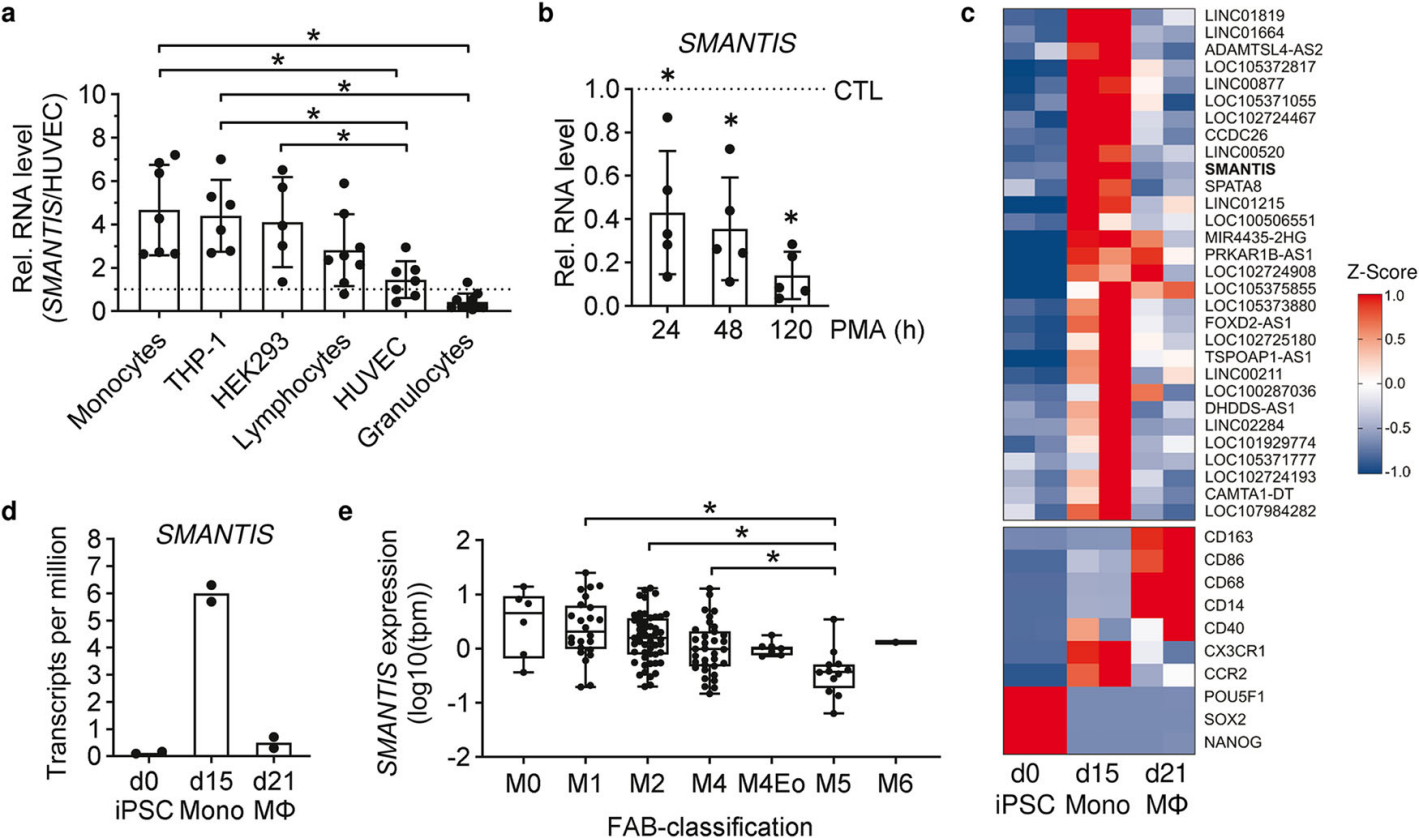
*SMANTIS* is highly expressed in monocytes. **a** *SMANTIS* expression in different cell types of peripheral blood mononuclear cells isolated by flow cytometry (granulocytes, *n* = 10; monocytes, *n* = 7; lymphocytes, *n* = 8) compared to cells cultured in cell culture (THP-1, *n* = 6; HUVEC, *n* = 7; HEK293, *n* = 5) as determined by RT-qPCR. Data was normalized to HUVEC (dashed line). Ordinary one-way ANOVA with Tukey post hoc test. **b** *SMANTIS* expression during differentiation of THP-1 cells treated with PMA (20 nM) for 24 h, 48 h, and 120 h followed by RT-qPCR. As control (CTL), DMSO was used. *n* = 5, Paired t-test. **c** Heat map of non-coding RNAs expressed by induced pluripotent stem cells (iPSC) differentiated to monocytes (Mono) and macrophages (M$\varphi$) at day 0 (d0), day 15 (d15), and day 21 (d21) as determined by RNA-Seq. The top30 altered ncRNAs are shown (up). Marker genes for iPSC (SOX2, NANOG, POU5F1), monocytes (CCR2, CX3CR1), and macrophages (CD40, CD14, CD68, CD163) show clustering of cells. Z-Score is shown on the right. *n* = 2. **d** *SMANTIS* expression in transcripts per million (tpm) as determined by RNA-Seq from iPSC (d0) induced differentiation to monocytes (d15), and macrophages (d21). **e** Acute myeloid leukemia (AML) subtypes and the respective *SMANTIS* expression calculated from a patient (>100) cohort (ID EGA: EGAD00001008484) shown as Box and Whiskers plot. Brown-Forsythe and Welch ANOVA with Games-Howell post hoc test. FAB, French-American-British classification; M0, minimally differentiated AML, *n* = 6; M1, AML without maturation, *n* = 24; M2, AML with maturation, *n* = 51; M4, myelomonocytic leukemia, *n* = 34; M4Eo, myelomonocytic leukemia with eosinophilia, *n* = 7; M5, monoblastic/monocytic leukemia, *n* = 12; M6, erythroleukemia, *n* = 1. Error bars are mean +/− SD. *p < 0.05.

Previously, the lncRNA *SMANTIS* was reported to be essential for endothelial cells, in particular the adhesion to monocytes^6^. A screening for the expression of *SMANTIS* in blood cells and selected cell lines revealed that *SMANTIS* was higher expressed in monocytic cells compared to all other tested cell types (Fig. 1a). THP-1, an accepted human leukemia monocytic cell line^15^, showed similar expression levels of *SMANTIS* compared to monocytes isolated from blood after FACS sorting (Fig. 1a, Supplementary Fig. 1a). Therefore, THP-1 cells were selected to reveal the function of *SMANTIS* in blood cells. Interestingly, when differentiating THP-1 into macrophage-like cells with Phorbol-12-myristat-13-acetate (PMA), *SMANTIS* expression was decreased after only 24 h (Fig. 1b). To investigate whether *SMANTIS* is specifically expressed in monocytes, and not macrophages, RNA-seq was performed after differentiation of inducible pluripotent stem cells (iPSC) into monocytes or further into macrophages (Supplementary Data 1-3). RNA-seq revealed that *SMANTIS* was induced after iPSC-differentiation to monocytes (d15), but lost again in macrophages (d21) (Fig. 1c, d, Supplementary Data 1-3), and that *SMANTIS* isoform NR_164153.1 was predominantly expressed in monocytes (Supplementary Fig. 1b). *SMANTIS* was one of the top altered lncRNAs expressed in monocytes compared to iPSC or macrophages (Fig. 1c). Since *SMANTIS* is a disease-relevant lncRNA as shown previously in endothelial cells isolated from glioblastoma and pulmonary arterial hypertension^5,6^, the clinical relevance of *SMANTIS* in blood cells was evaluated in a transcriptomic data set of an AML patient (>100) cohort^16^. After classification of the AML patients into the seven French-American-British (FAB) subtypes of AML, differences in *SMANTIS* expression could be observed across the different subtypes of AML (Fig. 1e, Supplementary Data 4). Based on the potential relevance of *SMANTIS* in monocytic cells as well as the diverse expression in AML patients, the molecular and functional relevance of *SMANTIS* was clarified more in detail.

### *SMANTIS* interacts with the transcription factor RUNX1

**Fig. 2:**
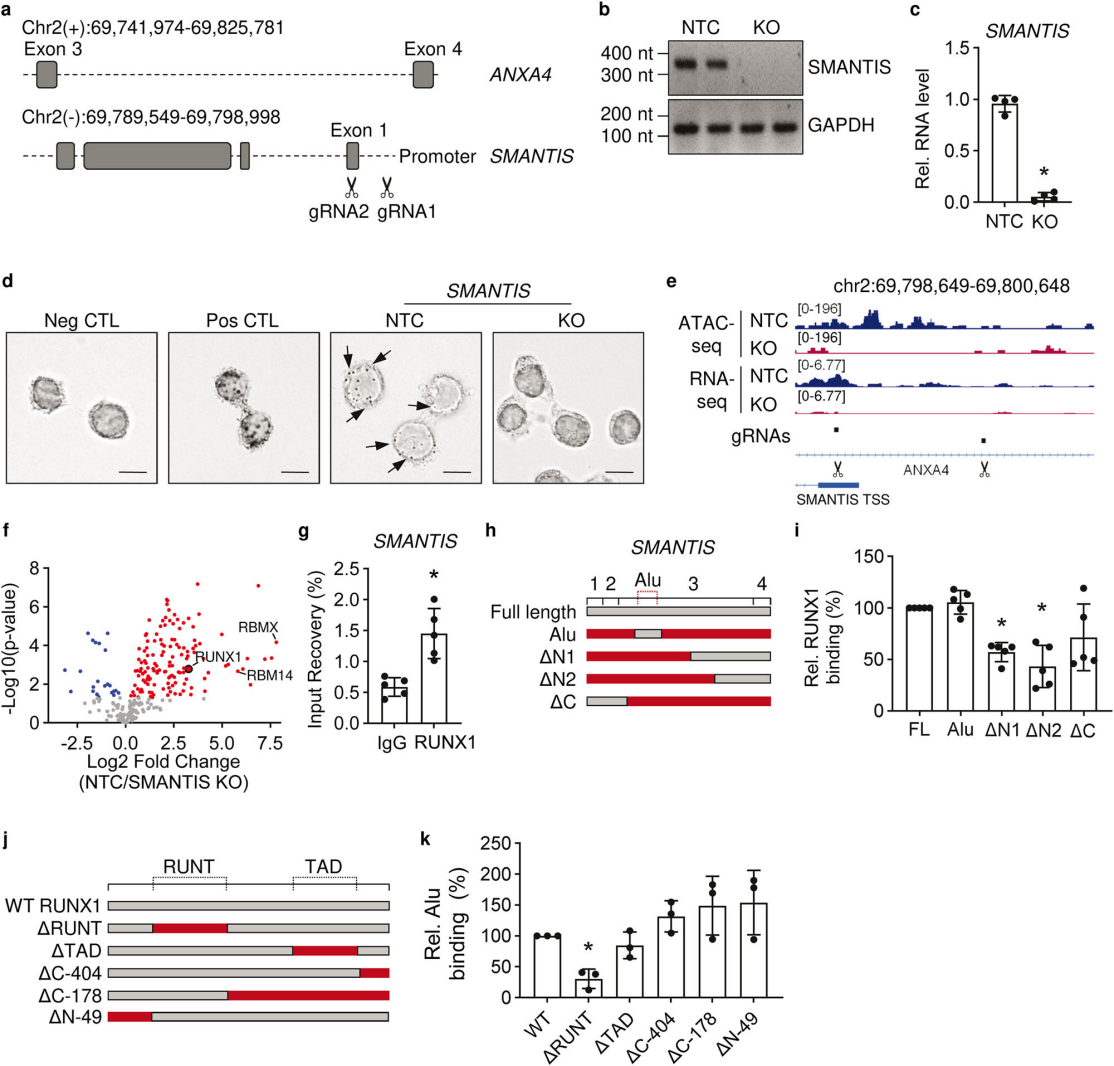
*SMANTIS* interacts with RUNX1. **a** Genomic structure and LentiCRISPRv2-mediated knockout strategy of *SMANTIS*. The combination of gRNA1 and gRNA2 was used for CRISPR/Cas9 of *SMANTIS* in THP-1 cells. gRNA, guide RNA. **b** Agarose gel electrophoresis after PCR of the genomic DNA region of *SMANTIS*. NTC, non-targeting control gRNA. GAPDH serves as loading control. **c** *SMANTIS* expression with and without CRISPR/Cas9 mediated KO as determined by RT-qPCR. *n* = 4, Mann-Whitney test. **d** RNA in situ hybridization of THP-1 cells with (KO) and without (NTC) CRISPR/Cas9 mediated KO of *SMANTIS* with RNAscope. Arrows indicate prominent *SMANTIS* expression. Representative images are shown. Scale bar indicates 10 µm. **e** IGV genome browser view of *SMANTIS* transcription start site (TSS) with tracks for RNA-Seq and Assay for Transposase-Accessible Chromatin (ATAC) of THP-1 cells with *SMANTIS* KO compared to control (NTC). The ATAC-seq signal in the KO is strongly reduced indicating successful gRNA-mediated deletion (scissors). **f** Volcano plot of RNA antisense-pulldown with oligonucleotides targeting *SMANTIS* in NTC versus *SMANTIS* KO THP-1. Red dots indicate protein interactions of *SMANTIS*, which are lost after *SMANTIS* KO. The negative log-transformed *p* values are shown on the y-axis. *n* = 4, students t-test. **g** RNA-immunoprecipitation (RIP) with antibodies against RUNX1 or IgG followed by RT-qPCR of *SMANTIS*. *n* = 5, paired t-test. **h**, **i** Scheme of *SMANTIS* mutants used (**h**) for the in vitro binding assay with recombinant RUNX1 followed by RT-qPCR (**i**). *n* = 5, One-way ANOVA with Dunnett’s post hoc test. Numbers indicate exon number; FL, full length; A, Alu element; N, N-terminus; C, C-terminus. **j**, **k** Scheme of RUNX1 mutants (**j**) used for the in vitro binding assay with the Alu element of *SMANTIS* followed by RT-qPCR (**k**). *n* = 3. One-way ANOVA with Dunnett’s post test. RUNT, DNA-binding domain; TAD, transcription activation domain. Error bars are mean +/− SD. *p < 0.05. KO, knockout.

To identify the functional relevance of *SMANTIS*, a *SMANTIS* LentiCRISPRv2-mediated knockout in THP-1 cells was generated. The knockout was achieved by removing the transcriptional start site (TSS), including adjacent parts of the promoter and of the first exon of *SMANTIS*, with two guide RNAs (gRNAs) (Fig. 2a). The successful knockout was validated by PCR with primers against the deleted genomic region and Sanger sequencing (Fig. 2b, Supplementary Fig. 1c, Supplementary Fig. 2), with RT-qPCR on cDNA level (Fig. 2c) and did not affect Anxa4 protein levels or splicing of *ANXA4* (Supplementary Fig. 3). Furthermore, RNA in situ hybridization using RNAscope targeting *SMANTIS* in THP-1 revealed that the localization of *SMANTIS* is predominantly nuclear, but, as expected, lost in *SMANTIS* knockout cells (Fig. 2d). An additional RNA-seq and Assay for Transposase-Accessible Chromatin (ATAC-seq) of *SMANTIS* knockout and non-targeting control (NTC) THP-1 cells revealed loss of *SMANTIS* expression accompanied by a loss of accessible chromatin of the deleted region (Fig. 2e, Supplementary Fig. 2). The data demonstrate a successful CRISPR-mediated knockout of *SMANTIS* in THP-1.

To gain insights into the molecular mechanism by which *SMANTIS* functions, we sought to identify its protein interaction partners. Therefore, an antisense pulldown with biotinylated antisense oligonucleotides targeting *SMANTIS* followed by mass spectrometry was performed. NTC cells were used as a reference to identify protein interaction partners, which were lost in *SMANTIS* knockout cells (Fig. 2f, Supplementary Data 5). *SMANTIS* interacted with many nuclear proteins, such as RBMX and RBM14 (Supplementary Data 5). Interestingly, the transcription factor RUNX1, which is important in the course of AML^17^, was found as an interaction partner of *SMANTIS* (Fig. 2f). Due to the previous findings on *SMANTIS* expression differences in the subtypes of AML, we directed our research towards RUNX1. RNA immunoprecipitation in THP-1 and human umbilical vein endothelial cells (HUVEC) with antibodies against RUNX1 demonstrated that *SMANTIS* interacted with RUNX1, but not with the IgG negative control or in HEK293T cells (Fig. 2g, Supplementary Fig. 4).

To reveal which part of *SMANTIS* is interacting with RUNX1, an in vitro binding assay of different in vitro transcribed *SMANTIS* mutants with recombinant RUNX1 protein was performed (Fig. 2h, i). Interestingly, a *SMANTIS* mutant containing only the Alu-element region was sufficient to interact as strongly as the full-length *SMANTIS* RNA with RUNX1, whereas loss of the Alu-element in N- or C-terminal deletions of *SMANTIS* strongly decreased the binding (Fig. 2i). To determine which part of RUNX1 is required for the interaction, we generated 6x-His tagged RUNX1 mutants either without important domains (RUNT, TAD) or with N- or C-terminal deletions (Fig. 2j, Supplementary Data 6). An in vitro binding assay of in vitro translated RUNX1 or RUNX1 mutants with His-Tag antibodies revealed that the RUNT domain, which is also important for the binding of the repetitive region of lncRNA LOUP to DNA^12^, is required for the interaction of RUNX1 with the Alu-element of *SMANTIS* (Fig. 2k). Our data suggest that *SMANTIS* and RUNX1 interact primarily through an Alu-RUNT interaction.

### *SMANTIS* and *RUNX1* regulate common target genes in THP-1 cells

**Fig. 3:**
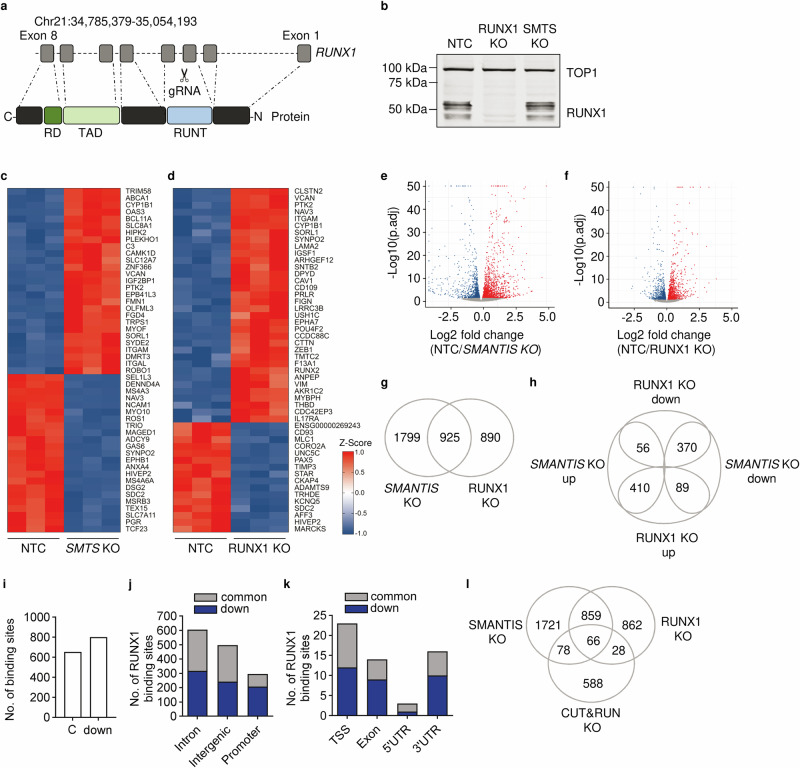
*SMANTIS* KO and RUNX1 KO regulate common target genes. **a** Scheme of the CRISPR/Cas9 KO strategy for RUNX1 in THP-1 cells by targeting a region next to exon 3 with a single gRNA. **b** Western blot analysis with antibodies against RUNX1 and topoisomerase I (TOP1) of control (NTC), RUNX1 knockout (KO), and *SMANTIS* KO (SMTS KO) in THP-1 cells. Heat map using Z-score of the top 50 differential regulated genes as determined by RNA-seq with (KO) and without (NTC) CRISPR/Cas9-mediated KO of *SMANTIS* (**c**) and RUNX1 (**d**). *n* = 3. **e**, **f** Volcano plots of the differential regulated genes from *SMANTIS* KO and RUNX1 KO compared to NTC. Log2 fold changes between NTC and the KO are shown on the x-axis, the negative logarithmic p-adjusted value (p.adj) is shown on the y-axis. **g** Venn diagram of the overlapping differentially regulated genes as determined by RNA-seq of *SMANTIS* KO and RUNX1 KO. **h** Comparison of the common up- and down-regulated genes between *SMANTIS* KO and RUNX1 KO. **i** Comparison of the changed downregulated accessible peaks (down) after RUNX1 CUT&RUN. Common (C; (NTC and *SMANTIS* KO)) were not changed. **j**, **k** Genomic localization of the RUNX1 binding sites in the genome of common and *SMANTIS* KO *(*down*)* specific binding sites. TSS, transcriptional start site; UTR, untranslated region. **l** Venn diagram of overlapping differentially expressed genes from *SMANTIS KO* and RUNX1 KO compared to CUT&RUN downregulated peaks in *SMANTIS* KO. KO, knockout.

Next, the potential influence of *SMANTIS* on RUNX1 was investigated. In addition to the *SMANTIS* knockout, a lentiCRISPR-mediated knockout of *RUNX1* was also generated in THP-1 cells. Therefore, a single gRNA approach with a gRNA targeting the splice site of exon 3 (RUNT domain) of *RUNX1* was used (Fig. 3a). This strategy led to a knockout of RUNX1 on protein level, while the *SMANTIS* knockout itself had no effect (Fig. 3b, Supplementary Fig. 5a, b). To determine the differential gene expression profile of *SMANTIS* and RUNX1 knockout THP-1 cells, RNA-seq was performed (Fig. 3c–f, Supplementary Data 7, Supplementary Data 8). The top 50 differentially expressed genes (Fig. 3c, d) revealed common genes affected by *SMANTIS* and RUNX1 knockouts. For example, *ITGAM* (known as CD11b), *VCAN* (known as Versican), and *PTK2* (known as Focal Adhesion kinase FAK), which were upregulated in *SMANTIS* and RUNX1 knockouts (Fig. 3c, d), are all associated with cell adhesion^18–20^. Additionally, in *SMANTIS* knockout cells, expression levels of additional cell adhesion associated genes such as *ITGAL*, *NCAM1*, and *EPHB1* and transcription factors such as *BCL11A* and *HIVEP2* were changed (Fig. 3c). Interestingly, knockout of RUNX1 showed altered gene expressions of transcriptional activators such as *AFF3* (Fig. 3d), present in super elongation complexes and a gene found to be fused with both RUNX1 and MLL in acute leukemia^21^, and of the bone lineage-specific transcription factor *RUNX2* (Fig. 3d), which is known to potentially compensate antitumor effects after RUNX1-silencing^22^. In total, 925 common differentially expressed genes were identified between *SMANTIS* and RUNX1 knockouts (Fig. 3g), with 410 commonly upregulated and 370 commonly downregulated (Fig. 3h). These findings suggest a complex regulation of gene expression by both knockouts with potential implications for leukemia and cell adhesion.

Given that RUNX1 is a transcription factor displaying DNA-binding activity^17^, we explored whether the binding of RUNX1 to its target genes is dependent on *SMANTIS*. CUT&RUN (Cleavage Under Targets & Release Using Nuclease) with RUNX1 antibodies followed by DNA sequencing was conducted to investigate the binding of RUNX1 to its target genes. CUT&RUN followed by high-resolution mapping of DNA binding sites affected by *SMANTIS* knockout showed that more than the majority of RUNX1-binding sites was decreased: 653 unaffected RUNX1-binding sites between control and *SMANTIS* knockout were found, but 801 RUNX1-binding sites decreased in *SMANTIS* knockout cells (Fig. 3i, Supplementary Data 9). Most of the decreased RUNX-1 binding-sites were located in intronic, intergenic, and promoter regions (Fig. 3j, k). Considering the difference between *SMANTIS* KO-decreased and unaffected (common) RUNX1 binding sites, the promoter regions were most strongly influenced (Fig. 3j). The *SMANTIS*-dependent alteration in RUNX1-DNA binding sites identified 66 genes in total that also exhibited differential expression in our RNA-seq analysis (Fig. 3l, Supplementary Data 7, Supplementary Data 9). To support our data, a motif enrichment analysis of the RUNX1 CUT&RUN data was performed with HOMER^23^. The motif results for the common and downregulated RUNX1 peaks showed that the RUNX motif was the top hit in each of the peaksets, although there is a difference in the percentage of peaks with the motif between the common and downregulated groups (common >60% of the peaks have the motif, and downregulated >30%) (Supplementary Fig. 6a, b). As *SMANTIS* knockout does not affect the protein abundance of RUNX1 (Fig. 3b), these data demonstrate that the DNA-binding behaviour of RUNX1 is partially dependent on lncRNA *SMANTIS*.

### *SMANTIS* and RUNX1 limit the cell adhesion of THP-1 monocytes to endothelial cells

**Fig. 4:**
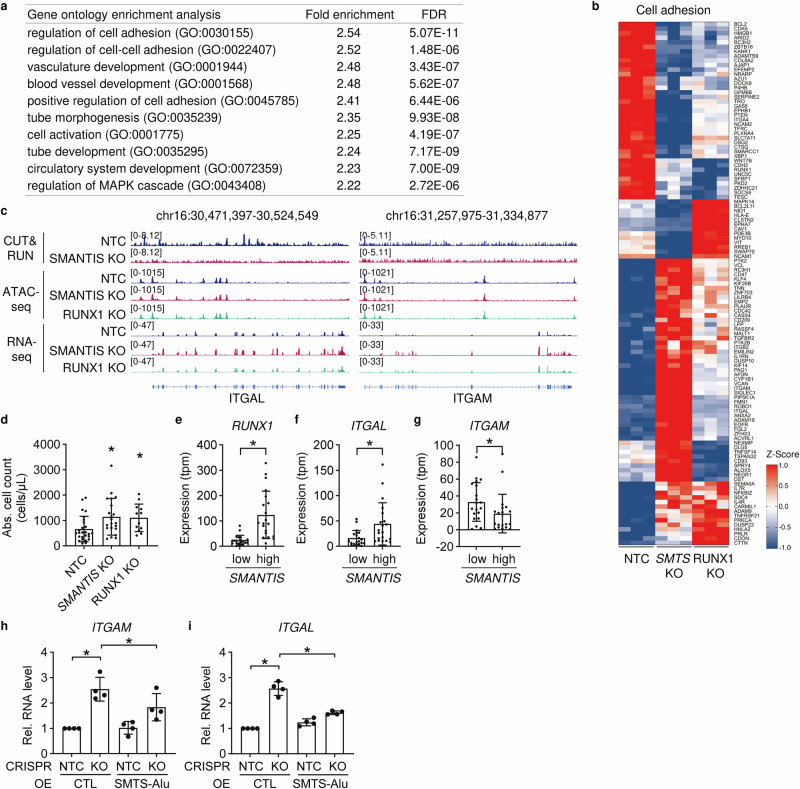
*SMANTIS* and RUNX1 limit adhesion of monocytes to endothelial cells. **a** Gene ontology (GO) enrichment analysis determined with PANTHER showing pathways associated with common differentially regulated genes from *SMANTIS* KO and RUNX1 KO compared to control NTC. The top 10 GO terms after fold enrichment are shown. FDR, false discovery rate. **b** Heat map of the differentially regulated genes using Z-score associated with regulation of cell adhesion determined by RNA-Seq. Comparison of NTC, *SMANTIS* (*SMTS*) KO, and RUNX1 KO. **c** IGV genome browser view of the loci of ITGAL and ITGAM after CUT&RUN for RUNX1, ATAC-seq and RNA-Seq for NTC, *SMANTIS* KO and RUNX1 KO. Numbers in brackets indicate the number of reads. **d** Flow cytometry based cell adhesion assay of NTC (*n* = 26), *SMANTIS* KO (*n* = 21), and RUNX1 KO (*n* = 12) cells attached to HUVEC. Ordinary one-way ANOVA with Holm-Sidak post hoc test. **e**, **f**, **g** Correlation of RUNX1 (**e**), ITGAL (**f**), and ITGAM (**g**) expression in AML-patients expressing the lowest (*n* = 20) or highest (*n* = 20) *SMANTIS* levels (tpm) from EGA dataset EGAD00001008484. Mann-Whitney test. **h**, **i** RT-qPCR of *ITGAM* (**h**) or *ITGAL* (**i**) after overexpression of the *SMANTIS-Alu* element or an empty pcDNA3.1+ vector (CTL) in NTC or *SMANTIS* KO THP-1 cells. NTC overexpressing CTL were set to 1. One-Way ANOVA with Tukey’s post-hoc test. *n* = 4. Error bars are mean +/− SD. *p < 0.05. KO, knockout.

To identify pathways that were affected by *SMANTIS* and RUNX1 knockouts, Gene Set Enrichment Analysis (GSEA) of the significantly regulated genes associated with *SMANTIS* and RUNX1 knockouts was performed. GSEA revealed predominantly genes associated with the regulation of cell adhesion (Fig. 4a). Genes associated with GO terms related to cell adhesion show altered gene expression between control (NTC), *SMANTIS*, and RUNX1 knockout (Fig. 4b). Among the top differential upregulated genes in *SMANTIS* knockout were *ITGAL* (Integrin Subunit Alpha L, also known as CD11a) and *ITGAM* (Integrin Subunit Alpha M, also known as CD11b) (Fig. 4c). ITGAL is part of lymphocyte function-associated antigen-1 (LFA-1), which is a receptor for the ICAM transmembrane protein family and important for lymphocyte-endothelial adhesion^24,25^. ITGAM enables binding of monocytes to IL-1 beta-stimulated endothelium^18^. Previously, an adhesion assay in HUVECs depleted from *SMANTIS* with siRNAs demonstrated increased endothelial-monocyte adhesion in an ICAM-1-dependent manner^6^. To determine the influence of *SMANTIS* and RUNX1 on monocyte adhesion to endothelial cells, a cell adhesion assay was conducted using *SMANTIS* or RUNX1 knockout THP-1 cells with wild-type HUVECs. Knockout of *SMANTIS* or RUNX1 increased the adhesion of THP-1 monocytes to endothelial cells (Fig. 4d). Additionally, the AML patients were categorized into 2 groups, the top 20 AML patients with the lowest *SMANTIS* expression and the top 20 AML patients with the highest *SMANTIS* expression. The AML patients with the lowest *SMANTIS* expression had significantly lower *RUNX1* and *ITGAL* (Fig. 4e, f, Supplementary Data 4), but higher *ITGAM* expression (Fig. 4g, Supplementary Data 4). Interestingly, overexpression of the Alu-element of *SMANTIS* in *SMANTIS* knockout THP-1 cells rescued the expression of *ITGAM* and *ITGAL* (Fig. 4h, i, Supplementary Fig. 6c). These data indicate that loss of *SMANTIS* either on the endothelial^6^ or the monocytic site (Fig. 4d) lead to increased adhesion of both cell types.

Since the osteoclast precursor marker ITGAM was upregulated in *SMANTIS* knockout, the role of *SMANTIS* during the differentiation of monocytes into osteoclasts was also examined. Therefore, *SMANTIS* or RUNX1 knockout THP-1 were differentiated into osteoclast-like cells under the constant stimulation of RANKL and M-CSF (Supplementary Fig. 7a), which was followed by RNA-seq (Supplementary Data 10, Supplementary Data 11). Before sequencing, osteoclast markers *ITGAM*, *ACP5*, and *MMP9* were tested with RT-qPCR and were significantly increased in osteoclast-like cells compared to basal THP-1 (Supplementary Fig. 7b). Expression levels of many ossification marker genes were increased in *SMANTIS* knockout compared to control and RUNX1 knockout (Supplementary Fig. 7c). However, the overlap of differentially expressed genes in *SMANTIS* and RUNX1 knockout THP-1-differentiated osteoclasts was limited (Supplementary Fig. 7d–g, Supplementary Data 10, Supplementary Data 11). These data indicate that RUNX1 and *SMANTIS* act in concert during cell adhesion of THP-1 monocytes to endothelial cells rather than during the monocytic differentiation to osteoclasts.

### *SMANTIS* mediates the interaction of RUNX1 with its accessory binding partners

**Fig. 5:**
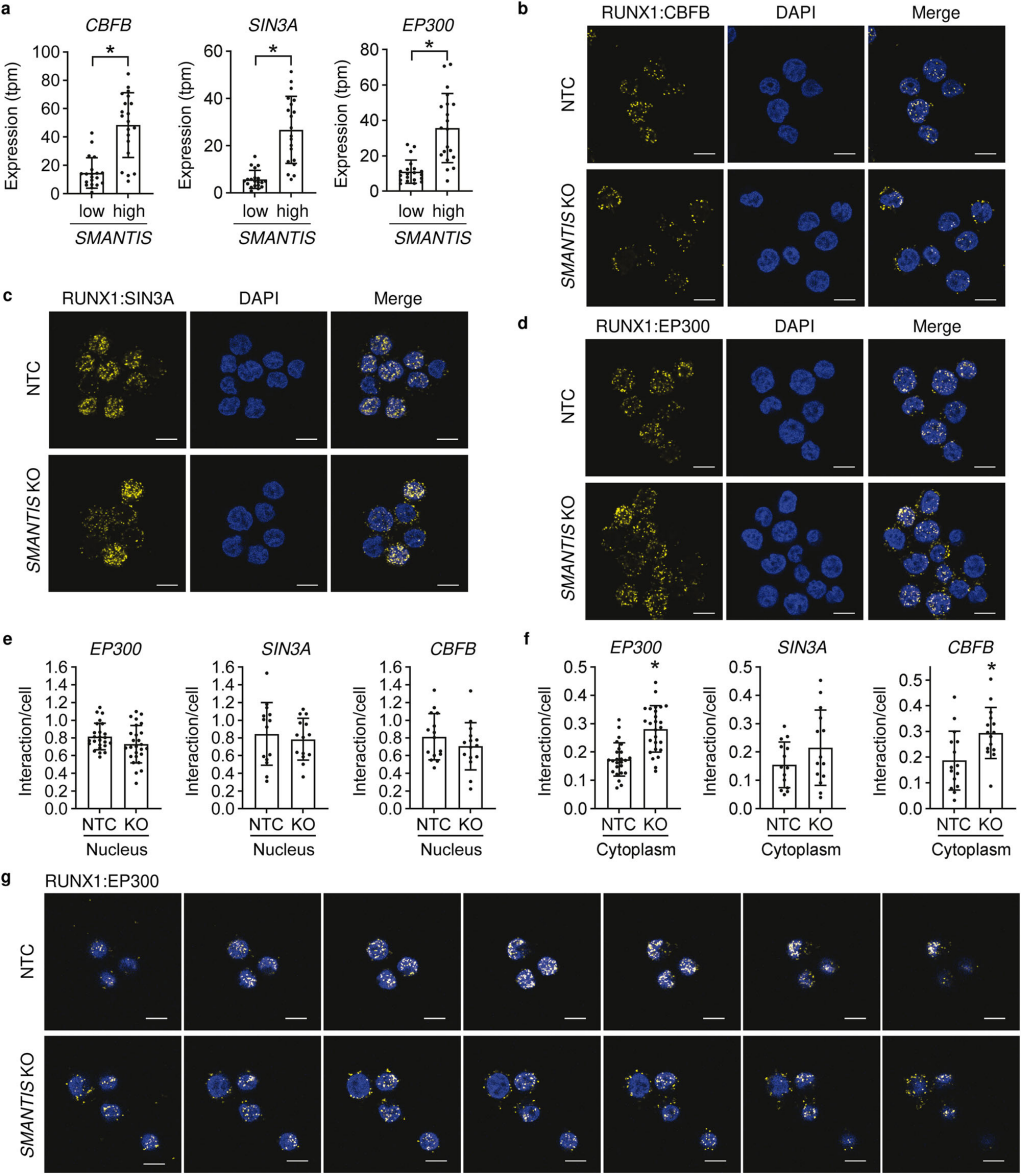
*SMANTIS* is required for the proper interaction of RUNX1 to the core-binding factor CBFB and the transcriptional co-activator protein EP300. **a** Correlation of RUNX1 interaction partners CBFB, SIN3A, and EP300 expression in AML-patients expressing the lowest (*n* = 20) or highest (*n* = 20) *SMANTIS* levels (tpm) from EGA dataset EGAD00001008484. Mann-Whitney test. **b**, **c**, **d** Proximity ligation assay (PLA) of RUNX1:CBFB (**b**), RUNX1:SIN3A (**c**) and RUNX1:EP300 (**d**) in NTC and *SMANTIS* KO THP-1 cells. Representative images are shown. Yellow dots indicate interactions. Nuclei are stained with DAPI. Scale bar indicates 10 µm. **e**, **f** PLA quantification of RUNX1:EP300 (NTC, *n* = 24; SMTS KO, *n* = 25), RUNX1:SIN3A (*n* = 14) and RUNX1:CBFB (*n* = 15) interactions in nucleus (**e**) and cytoplasm (**f**) of NTC and *SMANTIS* KO cells. *n* represents quantified images from in total three experiments. Mann-Whitney test. **g** Z-stack imaging of the RUNX1:EP300 interaction in NTC and *SMANTIS* KO cells determined with PLA. Representative Z-stacks are shown. Yellow dots indicate interactions. Nuclei are stained with DAPI. Scale bar indicates 10 µm. Error bars are mean +/− SD. *p < 0.05. KO, knockout.

RUNX1 is known to interact with other transcription factors, co-activators, and chromatin modulators to form activatory or repressive RUNX1-transcription factor complexes. Whereas Core-Binding Factor Subunit Beta (CBFB) is known as a stable co-factor for RUNX1, the interaction with the histone acetyltransferase and E1A Binding Protein P300 (EP300) serves activatory and the interaction with the transcriptional repressor SIN3A (SIN3 Transcription Regulator Family Member A) serves inhibitory transcriptionally purposes^26^. Since CBFB, SIN3A, and EP300 were all highly expressed in AML patients with higher *SMANTIS* expression values (Fig. 5a, Supplementary Data 4), we wondered whether *SMANTIS* is important for the recruitment of activatory or inhibitory co-factors of RUNX1. Proximity ligation assays of RUNX1 with CBFB, SIN3A, and EP300 in *SMANTIS* knockout cells revealed that the interaction of RUNX1 with EP300, SIN3A, and CBFB is not altered in the nucleus (Fig. 5b–e). However, the interaction of RUNX1 with CBFB and EP300 followed in many cells a ring-like nucleo-cytoplasmic pattern, and indeed, quantification of the number of interactions between RUNX1 and EP300 or CBFB was increased in the cytoplasm (Fig. 5f, g). Therefore, our data indicate that loss of lncRNA *SMANTIS* leads to altered localizations of RUNX1-EP300 or RUNX1-CBFB interactions.

## Discussion

**Fig. 6:**
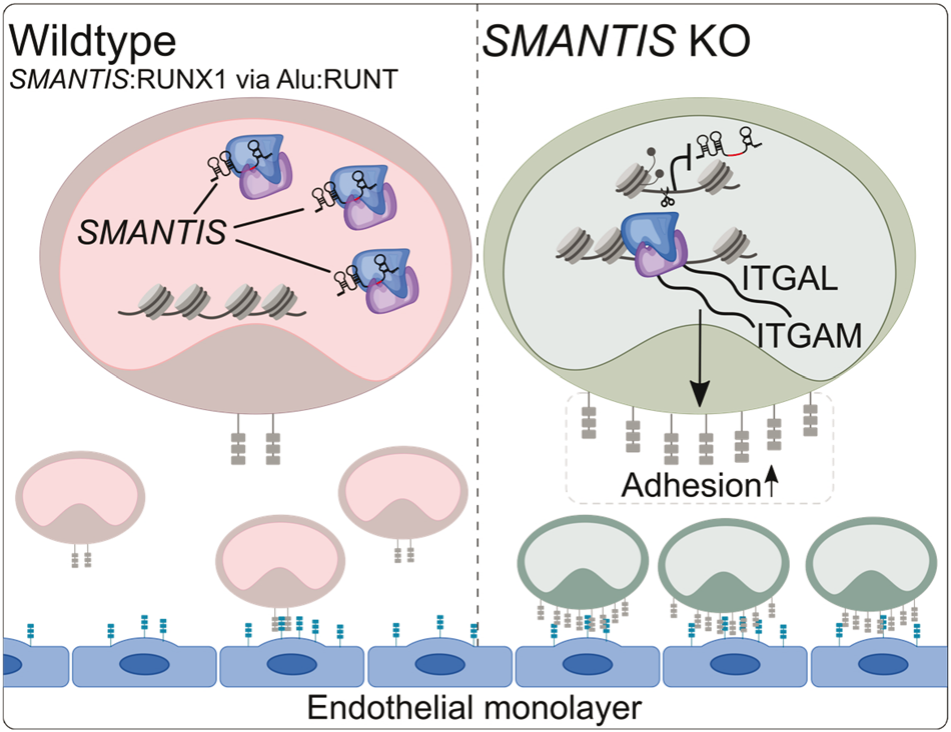
Molecular effects of lncRNA *SMANTIS* in THP-1 monocytes. *SMANTIS* interacts in the nucleus of monocytes with RUNX1 in a Alu-RUNT-dependent manner to control gene expression. In *SMANTIS* knockout (KO) cells, the interaction of *SMANTIS* and RUNX1 is lost, increasing the expression of the cell adhesion molecules *ITGAL* and *ITGAM* and the adhesion of THP-1 monocytes to endothelial cells. The model was drawn with Inkscape (v1.1.2).

The present study revealed that lncRNA *SMANTIS* is not only important for endothelial cells, but also for cell adhesion of monocytes to endothelial cells (Fig. 6), and its expression is changed in different AML subtypes. *SMANTIS* was higher expressed in monocytes compared to other immune cells, iPSC, endothelial cells, and differentiated macrophage-like cells. It interacted with transcription factor RUNX1, which is crucial for normal development of hematopoietic cell lineages and frequently mutated in AML^17^, through an Alu-RUNT dependent mechanism. Loss of *SMANTIS* altered the interaction of RUNX1 with its accessory binding partners CBFB and EP300. Interestingly, *SMANTIS* and RUNX1 shared common target genes and limited the regulation of cell adhesion to endothelial cells.

Enrichment analysis of the genes affected by *SMANTIS* and RUNX1 knockout revealed associations with the regulation of cell adhesion, which could be verified in attachment assays. Similarly, high and low expression levels of *SMANTIS* in the AML patient expression data were accompanied by differential expression levels of RUNX1, *ITGAM*, and *ITGAL* suggesting a role of *SMANTIS* in regulating the signalling pathways for adhesion. Therefore, *SMANTIS* might regulate multiple genes, probably through its Alu element, that are involved in cell adhesion pathways, expanding the current knowledge that *SMANTIS* decreases ICAM-1 in endothelial cells^6^.

The adhesion of monocytes to endothelial cells is important for several physiological and pathophysiological conditions. Once released from the bone marrow, classical monocytes only circulate for approximately a day before re-populating other tissues^27^. Here, *SMANTIS* might regulate the time-dependent adhesion and infiltration of monocytes into certain tissue, since it is induced in freshly differentiated monocytes and lost in further differentiated monocytes. Whether *SMANTIS* regulates specific surface markers or activates certain signalling pathways in monocytes that regulate which tissue to infiltrate remains to be investigated. Under pathophysiological conditions, in particular cancer, loss of *SMANTIS* might enhance the transendothelial migration of cancer cells and facilitate metastasis. Another relevant cardiovascular disease is atherosclerosis^28^ since *SMANTIS* limits adhesion in both monocytes (this study) and endothelial cells^6^.

Alu elements are transposable repetitive elements widely distributed in the human genome and involved in the regulation of gene expression^29^ and dictating enhancer–promoter selectivity^30^. Alu sequences have been proposed as a functional unit within lncRNA, e.g. as drivers of nuclear localization of long RNAs in human cells^31^. A study in myeloid cells revealed that lncRNA *LOUP* forms an active chromatin loop by recruiting RUNX1 to the enhancer and promoter of *PU.1*, a mechanism which is inoperative in t(8;21) AML, where RUNX1 is fused to ETO^12^. For *SMANTIS*, it was previously shown that it is predominantly nuclear localized and that the Alu element is important for binding of BRG1 to BAF155^5^. Similarly to the mechanism of lncRNA *LOUP* binding to RUNX1^12^, this study demonstrated that the presence of the Alu element in *SMANTIS* facilitates primarily the binding to the RUNT domain of RUNX1. The RUNT domain is important for the binding of RUNX1 to DNA^26^, emphasizing the potential mechanistic relevance of *SMANTIS* in RUNX1 targeted gene expression. However, the loss of *SMANTIS*, which did not result in changes in protein expression of monocytic RUNX1 under cell culture conditions, reduced the binding of RUNX1 to its target genes which might be due to an altered interaction of RUNX1 to EP300 or CBFB. The localization of the binding increased in the cytoplasm, potentially also in nuclear-cytoplasmic border regions. Knockout of both, *SMANTIS* and RUNX1, revealed common target genes including genes relevant for haematopoiesis, osteogenesis, and adhesion. As demonstrated by the motif enrichment analysis, *SMANTIS* partially supports the binding of RUNX1 to its target genes since >30% of the decreased RUNX1-binding sites contained the RUNX1 binding motif in the *SMANTIS* knockout. However, the altered RUNX1 binding behaviour resulted in minor effects on gene expression assuming that other effector proteins are involved. Additionally, it should be considered that potential changes on the nuclear architecture and interchromosomal interactions could potentially lead to altered gene expression^32^, downstream or upstream of the RUNX1 binding site. Here, around 550 binding sites were located outside of promoter regions limiting the identification of other genes effected by *SMANTIS* and RUNX1.

AML is a genetically diverse disease with multiple gene mutations and rearrangement of chromosomes, leading to uncontrolled proliferation of hematopoietic precursor cells^33^ and immature myeloid cells in bone marrow and peripheral blood^34^. The therapeutic options and drug efficacies vary among the patients, demonstrating the importance to clarify the underlining molecular mechanisms, which results in a great scientific and clinical challenge^16^. Alongside RUNX1, CEBPA was also identified as *SMANTIS* interaction partner. Depletion of *SMANTIS* additionally led to differential gene expression of *NCAM1* and *BCL11A*. CEBPA^35^, NCAM1^36^, RUNX1^17^, and BCL11A^37^ were all identified as risk factors for AML. Further, it would be interesting if *SMANTIS* changes its protein interaction partners under pathophysiological conditions, such as AML. Here, the identified proteins in the proteomic AML subtypes dataset established from the AML patient cohort^16^ would be of special interest.

Collectively, we identified *SMANTIS* as an lncRNA that limits cell adhesion in monocytes, binds to RUNX1 and regulates proteins involved in AML. However, further investigations including in vivo studies are required to elucidate whether modulation of *SMANTIS* could be exploited as a potential clinical therapy.

## Material and Methods

### Materials

The following chemicals and concentrations were used for stimulation: Phorbol-12-myristat-13-acetate (PMA, P8139, Sigma-Aldrich, 20 nM). The following antibodies were used: Recombinant Anti-RUNX1/AML1 (ab240639, Abcam), Anti-RUNX1 (AF2399, R&D Systems, for PLA), Anti-TopI (sc5342, Santa Cruz), Anti-P300 (A300358A, Bethyl), Anti-SIN3A (C15410250, Diagenode), Anti-CBFB (ab125191, Abcam), Anti-ANXA4 (sc-46693, Santa Cruz) and Anti-His tag (sc-8036, Santa Cruz Biotechnology).

### Cell culture

The human monocytic cell line THP-1 (from ATCC, LGC Promochem, Wesel, Germany, cat. No. TIB-202) was cultured in RPMI medium 1640 (61870-010, Gibco/Lifetechnologies, USA) supplemented with GlutaMAX, 8% heat-inactivated fetal calf serum (FCS) (F7524, Sigma-Aldrich) and 0.5% penicillin/streptomycin (P/S) (15140-122, Gibco/Lifetechnologies, USA) in a humidified atmosphere of 5% CO_2_ at 37 °C. THP-1 were regularly tested for mycoplasma contamination using the Venor GeM OneStep Mycoplasma detection kit for conventional PCR (11-8025, MB minerva biolabs, Germany).

Pooled human umbilical vein endothelial cells (HUVEC) purchased from PromoCell (C12203, Lot number 474Z010, 471Z011, 466Z022, Heidelberg, Germany) were cultured on precoated dishes with 0.2% gelatin (G1890, Sigma-Aldrich, Germany) in a humidified atmosphere of 5% CO2 at 37 °C. Endothelial growth medium kit enhanced (EGM) (PB-C-MH-100-2199, PeloBiotech, Germany) without hydrocortisone supplemented with 8% FCS, 0.5% P/S, and growth factors (EGF, bFGF, IGF, VEGF, Heparin, L-Glutamin) was used to culture HUVEC. HUVEC from passage 3 was used for the experiments.

Human embryonic kidney 293 cells (HEK293) were purchased from ATCC and cultured in DMEM High glucose Glutamax (31966-021, Gibco/Lifetechnologies, USA) and supplemented with Gentamicin (50 mg/ml) (15750-045, Thermo Fisher Scientific).

Human induced pluripotent stem cells (iPSC, WSTLi081-A, EbiSC, male) were kindly provided by Jaya Krishnan (Goethe University, Frankfurt, Germany) and were used for the iPSC differentiation to monocytes. iPSC were cultured as described in STEMdiff Monocyte Kit purchased from STEMCELL Technologies (#05320, STEMdiff^TM^ Monocyte Kit).

### Isolation of human peripheral blood mononuclear cells and granulocytes

Human peripheral blood mononuclear cells (PBMC) and granulocytes were isolated from commercially available buffy coats of anonymous donors (DRK Blutspendedienst Baden-Württemberg-Hessen, Institut für Transfusionsmedizin und Immunhämatologie, Frankfurt, Germany).

Isolation of PBMC and granulocytes was performed as described previously in ref. ^38^ with following adjustments. Briefly, 25 ml of blood was mixed with ACD (citric acid, sodium citrate, dextrose). Subsequently, 6% dextran with 0.9% natrium chloride (NaCl) was added and the mixture was inverted at least 18 times. After separation of the mixture into four 15 ml falcons (11 ml per falcon) and incubation for one hour at room temperature (RT), the yellowish supernatant was extracted from the settled blood and centrifuged at 1200 rpm for 12 min at 4 °C with brake on. 0.6 M calcium chloride (KCl) was added. The solution was subsequently adjusted to 50 ml with PBS (14190-094, Gibco/Lifetechnologies, USA) and centrifuged at 1300 rpm for 6 min at 4 °C using a high brake. The washing step was repeated until no red blood cells remained. The pellet was resuspended in PBS and carefully applied over 3 ml Ficoll (Pancoll human, P04-60500, PAN-Biotech, Germany) in a 15 ml tube and centrifuged at 1500 rpm for 30 min at 4 °C using a low brake. The PBMC layer and the granulocytes were separated and resuspended in FACS buffer (1% BSA, 2 mM EDTA). Cells were directly sorted with flow cytometry.

### Flow cytometry

For blood cells sorting, the isolated PBMC and granulocytes were separately analysed by using SH800S Cell Sorter from Sony Biotechnology. Cells were sorted based on their size indicated by forward scatter (FSC) and granularity indicated by sideward scatter (SSC) using the Cell Sorter Software Version 2.1.6 (Sony Biotechnology). Subsequently, cells were centrifuged at 1200 rpm for 4 min and resuspended in lysis buffer from the RNA Mini Kit (Bio&Cell). The software FlowJo version 10.7.1 was used for the analysis.

### RNA isolation, reverse transcription, and RT-qPCR

Total RNA was isolated and purified using the RNA Mini Kit as described in the manufacturer’s protocol (Bio&Sell). Reverse transcription with purified RNA was performed using SuperScript III Reverse Transcriptase (Thermo Fisher), oligo(dT)23, and random hexamer primers (Sigma). The RT-qPCR was performed with ITaq Universal SYBR Green Supermix with ROX as reference dye (Bio-Rad, 1752125) in an AriaMX cycler (Agilent). Relative expression of human target genes was normalized to β-actin and calculated using the ∆∆Ct method with the AriaMX qPCR software Version 1.7 (Agilent). The following primer sequences (human) were used: β*-Actin*, forward 5’-AAA GAC CTG TAC GCC AAC AC-3’ and reverse 5’-GTC ATA CTC CTG CTT GCT GAT-3’; *SMANTIS*, forward 5’-AAC TCC TGC TCC AAA CTC ACT C-3’ and reverse 5’- CAG CAA AGC ATT CTG ATG AAG-3’; Integrin subunit alpha M/CD11b (*ITGAM*), forward 5’-GCC TTG ACC TTA TGT CAT GGG-3’ and reverse 5’-CCT GTG CTG TAG TCG CAC T-3’; Integrin subunit alpha L (*ITGAL*), forward 5’-GAA GAG TCC AGG CTT CTG TC-3’ and reserve 5’-GGA GGT CTG AGT CCT CAT TG-3’; SMANTIS-Alu, forward 5’-ATC TCC TGA CCT CGT GAT CC-3’and reverse 5’-GGC TTG GTT GCT TCC GTA TG-3’; Matrix metalloproteinase-9 (*MMP-9*), forward 5’-TGG CAC CAC CAC AAC ATC AC-3’ and reverse 5’-GCG ACA CCA AAC TGG ATG AC-3’; Tartrate-resistant acid phosphatase (*ACP5*), forward 5’-GAC CAC CTT GGC AAT GTC TCT G-3’ and reverse 5’- TGG CTG AGG AAG TCA TCT GAG TTG-3’.

### RNA immunoprecipitation (RIP)

RNA immunoprecipitation was performed as described previously^39^. Briefly, 3×10^6^ THP-1, HEK293T or HUVEC were washed in Hanks buffer, seeded on a 10 cm plate and placed on ice. For UV-crosslinking, cells were irradiated with 0.150 J/cm^2^ 254 nm UV light (BIO-LINK, BLX-254, Vilber). Cells were subsequently transferred into a 15 ml falcon, centrifuged at 1000 x *g* at 4 °C for 4 min and resuspended in buffer A (10 mM HEPES pH 7.6, 10 mM KCL, 0.1 mM EDTA pH 8.0, 0.1 mM EGTA pH 8.0, 1 mM DTT, protein inhibitor mix (PIM), and phenylmethylsulfonylfluoride (PMSF)). The cell suspension was incubated on ice for 15 min and subsequently incubated with a final concentration of 0.75% Nonidet P40 (NP-40) (74385, Sigma Aldrich). After centrifuagtion at 16000 x *g* for 1 min at 4 °C, the cell suspension was washed twice with buffer A. Nuclear pellet was lysed with buffer C (20 mM HEPES pH 7.6, 400 mM NaCl, 1 mM EDTA pH 8.0, 1 mM EGTA pH 8.0, 1 mM DTT, PIM, and PMSF) for 15 min on a shaker at 4 °C and centrifuged at 1600 x *g* for 5 min at 4 °C. 10% of the lysate were used as input. 2 µg of the respective antibody was pre-coupled to 50 µl protein A magnetic beads (Dynabeads Protein A, 10002D, Invitrogen by Thermo Fisher Scientific) in buffer C3 (20 mM HEPES pH 7.6, 200 mM NaCl, 1 mM EDTA pH 8.0, 1 mM EGTA pH 8.0, 1 mM DTT, PIM, and PMSF) for 1 h at RT. The antibody-coupled beads were then washed in 1 M NaCl and subsequently washed twice with buffer C3. The beads were then added to the nuclear lysate and incubated on a rotor at 4 °C over night. Elution of the RNA was performed with QIAzol (Qiagen) as described in ref. ^39^. After RNA purification (Qiagen) and reverse transcription, RT-qPCR was performed as described above.

### Assay for Transposase-Accessible Chromatin with sequencing (ATAC-seq)

ATAC-seq was performed based on the protocol from Buenrostro et al.^40^ and modified by Ackermann et al.^41^. Briefly, 50,000 THP-1 were used for the ATAC library preparation performed with the Illumina Tagment DNA Enzyme and Buffer Kit (20034198, Illumina). Cells were washed in 50 µl cold PBS and centrifuged at 1200 rpm for 4 min. The cell pellet was resuspended in 50 µl of cold lysis buffer (48.5 µl of resuspension buffer (10 mM Tris-HCl pH 7.5 (15567-027, Thermo Fisher Scientific), 10 mM NaCl, 3 mM MgCl_2_, 0.5 µl 10% NP-40, 0.5 µl 10% Tween-20 (P1379, Sigma-Aldrich), and 0.25 µl 2% Digitonin (G9441, Promega)) and incubated for 3 min on ice. Wash buffer (990 µl of resuspension buffer, 10 µl 10% Tween-20) was added to the cell lysate and centrifuged at 1000 x *g* at 4 °C for 5 min. The cell pellet was resuspended in transposition reaction mix (25 µl TD Buffer, 16.5 µl PBS, 0.5 µl 10% Tween-20, 0.25 µl 2% Digtonin, 2.5 µl Tn5, 5 µl H_2_O) and incubated at 1000 rpm at 37 °C for 30 min. DNA was purified by using MinElute PCR Purification Kit (Qiagen), eluated in 20 µl elution buffer, and stored at -20 °C until sequencing.

The amplification of library together with indexing primers was performed as described elsewhere^40^. Libraries were mixed in equimolar ratios and sequenced on NextSeq2000 platform using a P3 flowcell. ATAC-seq reads were aligned to the GRCh38 primary assembly genome using *Bowtie2* (v2.4.5^42^ with the parameter *very-sensitive)*. Duplicate reads were removed using *samtools rmdup* (v1.1.0)^43^, and normalized genome browser coverage tracks were produced from the aligned reads using *bamCoverage*^44^, normalized by reads per kilobase per million mapped reads (RPKM), and using an effective genome size of 2,864,785,220. ATAC-seq peaks were called per replicate using *MACS3* (v3.0.0)^45^ with the parameter –scale-to-small. BigWig files were used to visualize peaks in Integrative Genomics Viewer (IGV) (Version 1.16.2).

### RNA-seq

RNA and library preparation integrity were verified with LabChip Gx Touch 24 (Perkin Elmer). 0.3–1 µg of total RNA was used as input for SMARTer Stranded Total RNA Sample Prep Kit - HI Mammalian (Takara Bio). Sequencing was performed on the NextSeq2000 instrument (Illumina) with 1 x 72bp single end setup.

Trimmomatic version 0.39 was employed to trim reads after a quality drop below a mean of Q20 in a window of 20 nucleotides or Q15 in a window of 5 nucleotides and keeping only filtered reads longer than 15 nucleotides^46^. Reads were aligned versus Ensembl human genome version hg38 (Ensembl release 104 or 109) with STAR 2.7.10a^47^. Aligned reads were filtered to remove duplicates with Picard 2.25.5, or Picard 3.0.0, multi-mapping, ribosomal, or mitochondrial reads. Gene counts were established with featureCounts 2.0.2, or featureCounts 2.0.4 by aggregating reads overlapping exons on the correct strand excluding those overlapping multiple genes^48^. The raw count matrix was normalized with DESeq2 version 1.30.1 or version 1.36.0^49^. Contrasts were created with DESeq2 based on the raw count matrix. Genes were classified as significantly differentially expressed at average count > 5, multiple testing adjusted *p*-value < 0.05, and -0.585 < log2FC > 0.585.

Raw genomic data from AML patient cohort^16^ uploaded at the European Genome-Phenome Archive (EGA) with the study accession ID EGA: EGAS00001005950 were used and analyzed in this study. For the analysis of the transcriptomes of the AML patient cohort and the iPSCs, raw reads were aligned against the hg38 genome and quantified using Salmon (v1.5.2), with default parameters. All downstream analyses were based on raw counts normalized to transcripts per million (TPM)^50^. AML patient subtypes were classified with the French-American-British (FAB) system^51^.

Normalized gene expression values of differentially expressed genes were used for a number of downstream analyses. Counts were transformed to Z-scores for visualization as heatmaps, and adjusted *P* values were negative log-transformed and shown alongside fold change values in volcano plots. RStudio (R version 4.1.2) was used to create the heatmaps and volcano plots.

### RUNX1 Cleavage Under Targets & Release Using Nuclease (CUT&RUN), library preparation and sequencing

DNA binding sites of RUNX1 were determined by using the CUT&RUN method, originally established by Skene and Henikoff in 2017^52^, as described in the EpiCypher CUT&RUN Protocol v2.0 and in Boos et al.^39^ with modifications for cell type and antibody. Here, 500,000 NTC, *SMANTIS* KO, and RUNX1 KO THP-1 were used and first washed with wash buffer (20 mM HEPES pH 7.9, 150 mM NaCl, 500 nM spermidine 1X Roche Protein Inhibitor Cocktail) at RT. Cells were then resuspended in a wash buffer and immobilized on magnetic beads. Therefore, cells were incubated with 10 µl BioMag®Plus Concanavalin A (ConA) beads (Polysciences, 86057-3) for 10 min at RT. Beads were washed once on a magnetic rack in 100 µl antibody buffer (wash buffer, 0.25% Digitonin, and 2 mM EDTA) and 1 µl RUNX1 antibody (ab240639, Abcam). After an over-night incubation of the beads with antibody with gentle shaking at 4 °C, the beads were washed twice with 200 µl 0.25% Digitonin wash buffer followed by resuspension and incubation in Digitonin wash buffer containing 2 µl CUTANA™ pAG-MNase (15-1016, EpiCypher) on ice for 30 min. After washing the samples twice, the samples were resuspended in 100 µl Digitonin wash buffer with addition of 2 µl CaCl_2_ (final concentration of 100 mM) and incubated for 2 h at 4 °C with gentle shaking. To stop the enzymatic reaction, samples were incubated in 33 µl stop buffer (340 mM NaCl, 20 mM EDTA, 4 mM EGTA, 0.25% Digitonin, 100 µg/ml RNase A, 50 µg/ml Glycoblue) for 10 min at 37 °C. Samples were then placed on a magnetic rack and supernatant containing the CUT&RUN-enriched DNA was collected in a fresh 1.5 ml tube. DNA purification was performed by using 5X volume of binding buffer (20 mM HEPES pH 7.9, 20 mM KCl, 1 mM CaCl_2_, 1 mM MnCl_2_). The pH was adjusted with sodium acetate before transferring the sample into a purification column (58002, ActiveMotif). Samples were then centrifuged at 11,000 x *g* for 30 sec. The column was washed with 750 µl wash buffer and dried by centrifugation for 2 min. The DNA was eluted in 25 µl elution buffer and concentration was measured with Qubit 3.0 Fluormeter (Life Technologies).

For library preperation, DNA fragments were cleaned up by 1xSPRI bead cleanup and used as input for SMARTer ThruPLEX DNA-seq Kit (Takara Bio) following the manufacturer’s protocol with exception of individual library cleanup after final PCR. Libraries were mixed in equimolar ratios and sequenced on NextSeq2000 platform using P3 flowcell.

Paired end CUT&RUN sequencing reads were aligned to the GRCh38 primary annotation genome build using *Bowtie2* (v2.4.5)^42^ with the parameter *–very-sensitive*. Mates were fixed and duplicate reads removed using *samtools fixmate* and *samtools markdup –r*, respectively (v1.1.0)^43^. Reads mapping to blacklisted regions of the GRCh38 genome were removed using *bedtools intersect –v* (v2.27.1)^53^, with the blacklisted regions taken from ENCODE^54^. Peaks were called per replicate using *MACS3 callpeak*, and differential binding analysis was performed using *MACS3 bdgdiff* to compare read pile-up in peak regions between conditions using a log-likelihood ratio (MACS v3.0.0)^45^. The relative sequencing depths of each condition are also taken into account in this test. The genomic locations of differential CUT&RUN peaks were then computed using *HOMER annotatePeaks.pl* (v4.1.1)^23^, which returns the positions of peak regions relative to genes annotated in the hg38 genome. Normalized coverage tracks for visualization in a genome browser were generated from the normalized bedGraph output of *MACS3*, and were converted to bigWig using *bedGraphToBigWig* from the *kentUtils* collection of tools from UCSC.

### Motif enrichment analysis

Motif enrichment analysis was performed using HOMER (v4.11)^23^. Briefly, unchanged or downregulated RUNX1 CUT&RUN peak sequences were extracted from the GRCh38 genome assembly using bedtools (v2.27.1) getfasta^53^. These sequences served as input to findMotifs.pl from HOMER.

### Proximity ligation assay (PLA)

PLA was performed using the Duolink In Situ Detection Reagents Orange Kit (DUO92007, Sigma-Aldrich). Briefly, 250,000 THP-1 were seeded in 8-well µ-ibidi slides and fixed using 4% paraformaldehyde (PFA) for 30 min. Afterwards, the cells were permeabilized with 0.05% Triton X-100 for 10 min and subsequently blocked with Duolink Blocking Solution at RT for 60 min. Primary antibodies were diluted 1:500 in blocking buffer and added to the samples. Cells were incubated with the individual antibody combination overnight at 4 °C. The following combinations of primary antibodies were used: Anti-RUNX1(goat(-)):Anti-EP300(rabbit(+)), Anti-RUNX1(goat(-)):Anti-SIN3A(rabbit(+)), and Anti-RUNX1(goat(-)):Anti-CBFB(rabbit(+)). At the following day, cells were washed with 0.3% Tween-20 in PBS and incubated with the corresponding MINUS and PLUS PLA probes for 60 min at 37 °C. Cells were then washed with wash buffer A, incubated with ligation buffer at 37 °C for 30 min, washed with wash buffer A, incubated with amplification buffer at 37 °C for 100 min, washed with wash buffer B, and stained with DAPI (washing and incubation steps were performed according to the manufacturer’s instructions). For preservation, 0.02% sodium azide diluted in PBS was added to the cells. Fluorescent images were taken with a Zeiss LSM800 laser scanning microscope (Carl Zeiss Microscopy GmbH), acquired with the ZEN 3.2 (blue edition) software (Carl Zeiss Microscopy GmbH), and analysed with Image J-win.64 v1.54h (BioVoxxel, Germany).

### Protein isolation, nuclear extraction, and western blot by SDS-PAGE

THP-1 were washed twice with cold Hanks solution (Applichem) and lysed with buffer A for 15 min on ice. Following incubation, 10% NP-40 was added to the cells for a final concentration of 0.75%, vortexed, and centrifuged at 17,000 x *g* for 1 min. The supernatant (cytosol) was removed and the nuclei pellet was further resuspended with buffer C to extract the nuclear proteins. The pellet was then incubated in buffer C for 15 min on a shaker at 4 °C. Subsequently, nuclei lysate was centrifuged at 17,000 x *g*. Nuclear extract was collected in a new tube. The protein concentration was determined by the Bradford assay and extract was boiled with Laemmli buffer. Equal amounts of proteins were loaded and separated with SDS-PAGE. Following protein separation, gels were blotted onto a nitrocellulose membrane. Next, the membrane was blocked with Rotiblock (A151.3, Carl Roth, Germany) and incubated with the first antibody. After several washing steps with and without Tween-20, an respective infrared-fluorescent-dye-conjugated secondary antibody (Licor) was added to the membrane. The proteins on the membrane could then be determined by the fluorescent signal, which was detected with an infrared-based laser scanning detection system (Odyssey Classic, Licor, Bad Homburg, Germany). Images were analysed with the Image Studio Version 5.2.5 (Licor).

### RNAscope

RNAscope 2.5 HD Detection Reagent (322310, Advanced Cell Diagnostics (ACD), USA) is a single-plex chromogenic brown assay used to detect expressed gene targets. The assay was performed according to manufacturer’s instructions with minor changes as described in ref. ^5^ and adjusted to suspension cells. Briefly, 250,000 THP-1 were seeded on a 8-well µ-ibidi slides. After settling for 1 h, the cells were fixed with 4% PFA at RT for 30 min. Next, cells were permeabilized with 0.1% Tween-20 in PBS and subsequently incubated with hydrogen peroxide for 10 min at RT to block endogenous peroxide activity. Further, cells were incubated with Protease III (dilution 1:15), a moderate protease treatment specifically for cultured cells for 10 min at RT using the HybEZ System. Here, the *SMANTIS* probe Hs-AK125871 (483551, ACD) and the amplification solutions were applied according to the instructions. The cells were finally counterstained with 10% Gill’s Hematoxylin No. 1 for 15 sec and washed with tap water. For preservation, 0.02% sodium azide diluted in PBS were added to the cells. Cells were imaged with the fluorescence microscope BZ-X800 (Version 01.03.00.001, Keyence, Germany) and adjusted with the BZ-X800 Analyzer (Version 1.1.2.4, Keyence, Germany).

### Flow cytometry-based adhesion assay

The flow cytometry-based quantification of monocytes adhesion to endothelial cells (EC) was performed as previously described in Vincent et al.^55^ with the following modifications and adjustments. 300,000 HUVEC were seeded on a six-well plate to ensure 80-90% confluence on the following day. 500,000 THP-1 were stained with Vybrant Dil Cell-Labeling Solution (V-22885, Thermo Fisher Scientific) as described in the manufacturer’s instructions. Labeled THP-1 were resuspended in EGM and added to the 6-well plates containing HUVEC for 30 min. Unbound monocytes were subsequently removed by washing gently three times with media. To detach the cells, trypsin was added and cells were centrifuged and resuspended in FACS Buffer. The cell suspension was then incubated with counting beads and analysed by flow cytometry using the Cell Sorter Software Version 2.1.6 (Sony).

### LentiCRISPRv2

A dual guide RNA (gRNA) system approach was used to knockout *SMANTIS*. The design of the gRNAs was based on the web-interface of CHOPCHOP^56^. Here, gRNA-1 (5’-CAC CGG CCC CCA AGG AGC TAA CTG G-3’ and 5’-AAA CCC AGT TAG CTC CTT GGG GGC C-3’) targeted a region upstream of the transcription start site (TSS), whereas gRNA-2 targeted a region in the first exon (5’-CAC CGG GTC ACA CGA CCA GAT ATT G-3’ and 5’-AAA CCA ATA TCT GGT CGT GTG ACC C-3’). In terms of RUNX1 knockout, the gRNA design was based on using Benchling (2022; https://www.benchling.com/). A single gRNA (5’-CAC CGA CCT TGA AAG CGA TGG GCA-3’ and 5’-AAA CTG CCC ATC GCT TTC AAG GTC-3’) approach targeting the third exon was performed. For the non-target control (NTC) also a single gRNA approach was used (5’-CAC CGT TCC GGG CTA ACA AGT CCT-3’ and 5’-AAA CAG GAC TTG TTA GCC CGG AAC-3’).

The gRNAs were cloned into a lentiCRISPRv2 vector backbone via Esp3l as described in ref. ^57^. lentiCRISPRv2 was a gift from Feng Zhang (Addgene plasmid #52961; http://n2t.net/addgene:52961; RRID:Addgene_52961)^57^. Afterwards, the gRNA containing lentiCRISPRv2 vectors were sequenced and purified. Lentivirus production was performed by transfecting Lenti-X 293 T cells (632180, Takara) with polyethylenimine (PEI) (40872, Sigma-Aldrich), packaging plasmid psPAX2, and the envelope plasmid pVSVG (pMD2.G). psPAX2 and pMD2.6 were a gift from Didier Trono (12260, Addgene plasmid, http://n2t.net/addgene:12260; RRID:Addgene_12260 and 12259, http://n2t.net/addgene:12259; RRID:Addgene_12259, respectively). After 24 h and 48 h post-transfection, the supernatant containing virus was harvested, centrifuged and stored at −80 °C. The produced virus was transduced into THP-1 by two consecutive spinoculations as previously described in ref. ^58^. Briefly, 200,000 cells were pelleted and resuspended in 1 ml of non-concentrated lentiviral particles. Polybrene (8 µg/ml) was added to increase transduction efficiency. Cells were then centrifuged at 1000 x *g* for 90 min at RT. After spinoculation, cells were resuspended in fresh growth medium and incubated for 48 h. Then a second spinoculation was performed with the same cells. Selection of transduced cells was maintained by adding puromycin (2 µg/ml) for 10–14 days. Medium was exchanged every 1–3 days. To confirm positive clones in terms of *SMANTIS* knockout, cells underwent clonal expansion after selection. Deletion was confirmed by applying the PCR amplified genomic DNA amplicons on agarose gel electrophoresis. The gDNA was isolated by resuspending cells after washing with lysis buffer containing proteinase K and incubated for 45 min at 56 °C. Lysate was then centrifuged and pellet was washed with 70% ethanol. Finally, gDNA was eluted in TE-Puffer (pH 8.0). The following primers were used for amplifying the gDNA with PCR: *SMANTIS*, forward 5’-AAC TCC TGC TCC AAA CTC ACT C-3’ and reverse 5’-AAC ATT CCC GGC ACC CTG AG-3’; *GAPDH*, forward 5’-TGG TGT CAG GTT ATG CTG GGC CAG-3’ and reverse 5’-GTG GGA TGG GAG GGT GCT GAA CAC-3’. The RUNX1 knockout was confirmed with Western blot as described above.

### Differentiation of THP-1 to osteoclast-like cells

Differentiation of THP-1 to osteoclast-like cells was performed as previously described in Goettsch et al.^59^ with the following modifications. 400,000 cells were resuspended in α-Minimum Essential Medium (α-MEM) (12571-063, Gibco/Lifetechnologies, USA), 10% heat-inactivated FCS, 1% P/S, and 30 ng/ml M-CSF (300-25, PeproTech, Thermo Fisher Scientific, Germany) for 2 days. Subsequently, cells were cultured for 26 d in differentiation medium (α-MEM, 10% FCS, 1% P/S, 30 ng/ml M-CSF, and 50 ng/ml RANKL (310-01, PeproTech, Thermo Fisher Scientific, Germany)). The medium was changed every 3-4 d by centrifuging the non-adherent cells and reseeding the cells attached to the bottom of the well. After differentiation, RNA was isolated as described above. Prior to sending for RNA-seq, osteoclast marker genes were confirmed with RT-qPCR.

### Antisense-oligonucleotide pulldown of lncRNA *SMANTIS*

Antisense oligonucleotide pulldown was performed as described previously in Oo et al.^60^. Based on the RNA sequence of *SMANTIS*, the antisense oligonucleotides with a 5’-biotin tag were designed by using the online GeneGlobe tool (QIAgen). NTC and *SMANTIS*-KO THP-1 were washed with Hanks, crosslinked with UV-light on ice (0.150 J/cm^2^ 254 nm UV light (BIO-LINK, BLX-254, Vilber) and centrifuged. Subsequently, nuclei disruption was obtained by flash freezing and thawing the cell pellets. Cells were resuspended in 200 µl buffer L (50 nM Tris/HCl pH8, 50 mM NaCl, 0.5% NP-40, 1 mM EDTA, PIM, PMSF, DTT, and superase 1 µl/ml), incubated for 30 min on ice, and subsequently centrifuged at 10,000 *g* at 4 °C for 3 min. 1 ml buffer L and 20 µl myOne Streptavidin C1 beads (65001, Dynabeads MyOne Streptavidin C1, Thermo Fisher Scientific) were added to the lysate and incubated for 30 min at 4 °C for pre-clearing. Following the pre-clearing step, 200 pmol of *SMANTIS* antisense-oligonucleotide (5’-TGT GAA GCA GGC ACG TTG ATA-3’) were added to the lysate. Annealing of the antisense oligonucleotides to the RNA was enabled by rotating the mix overnight at 4 °C. Next, 50 µl MyOne Streptavidin C1 beads were added to the lysate and rotated for 2 h at 4 °C. The RNA-attached beads were then washed and used for mass spectrometry.

For mass spectrometry, beads were supplemented with 6 M GdmCl, 50 mM Tris/HCl, 10 mM TCEP and incubated at 95 °C for 5 min. Reduced thiols were alkylated with 40 mM chloroacetamid and samples were diluted with 25 mM Tris/HCl, pH 8.5, 10% acetonitrile to obtain a final GdmCl concentration of 0.6 M. Proteins were digested with 1 µg Trypsin (sequencing grade, Promega) overnight at 37 °C under gentle agitation. Digestion was stopped by adding trifluoroacetic acid to a final concentration of 0.5%. Peptides were fractionated on multi-stop-and-go tips (StageTips) containing C18-tips and strong cation exchange (SCX) tips^61^. Peptides were eluted in wells of microtiter plates and peptides were dried and resolved in 1% acetonitrile, 0.1% formic acid.

Liquid chromatography / mass spectrometry (LC/MS) was performed on Thermo Scientific™ Q Exactive Plus equipped with an ultra-high performance liquid chromatography unit (Thermo Scientific Dionex Ultimate 3000) and a Nanospray Flex Ion-Source (Thermo Scientific). Peptides were loaded on a C18 reversed-phase precolumn (Thermo Scientific) followed by separation on a 2.4 µm Reprosil C18 resin (Dr. Maisch GmbH) in-house packed picotip emitter tip (diameter 100 µm, 15 cm from New Objectives) using a gradient from 4% acetonitrile, 0.1% formic acid to 30% eluent B (99% acetonitrile, 0.1% formic acid) for 30 min and an additional gradient to 60% eluent B for 5 min with a flow rate 300 nl/min and washout with 99% B for 5 min.

MS data were recorded by data dependent acquisition. The full MS scan range was 300 to 2000 m/z with resolution of 70000, and an automatic gain control (AGC) value of 3*10E6 total ion counts with a maximal ion injection time of 160 ms. Only higher charged ions (2+) were selected for MS/MS scans with a resolution of 17500, an isolation window of 2 m/z and an automatic gain control value set to 10E5 ions with a maximal ion injection time of 150 ms. MS1 Data were acquired in profile mode.

The data processing protocol includes data analysis with MaxQuant 2.0.1.0^62^, Perseus 1.6.1.3^63^, and Excel (Microsoft Office 2016). N-terminal acetylation (+42.01) and oxidation of methionine (+15.99) were selected as variable modifications and carbamidomethylation (+57.02) on cysteines as a fixed modification. The human reference proteome set (Uniprot, August 2023, 104436 entries) was used to identify peptides and proteins with a false discovery rate (FDR) less than 1%. Reverse identifications and common contaminants were removed. Proteins were filtered to be at least identified 3 times (*n* = 4) in one experimental group. Missing values were replaced by lowest value of the data set. Significant interacting proteins were determined by student´s t-test (p-value) and permutation based FDR (q-value). Volcano plot of the identified interacting proteins was performed with RStudio (R version 4.1.3) using negative log-transformed *P* values and shown alongside fold change values in volcano. Proteins identified containing more than one peptide were shown in Supplementary Data 5.

### Cloning of *SMANTIS* mutants, in vitro transcription of *SMANTIS* constructs, and in vitro binding assay

*SMANTIS* mutant constructs originated from Leisegang et al.^5^. In brief, the *SMANTIS*-Alu element contruct (pcDNA3.1+*SMANTIS*-Alu) was generated by PCR amplification of the Alu-contaning fragment with 5’-ATT TGG TAC CCA AAT GTC TTC AAG GTA TAA AAA TGT GGT CTA-3’ and 5’- ATT TGG GCC CGT GCT GGA TGC AGA TAA TGT TTG ACT CCT-3’. After digestion with Acc65I/ApaI, the fragment was ligated into pcDNA3.1+. For the other mutants, the full length pcDNA3.1 + *SMANTIS* was digested either with XbaI/Acc65I (for ΔN1), with BlpI/Acc65I (for ΔN2) or with Eco47III/ApaI (for ΔC). Afterwards, Klenow Fill-In (for ΔN1 and ΔN2) or 3’-5’ exonuclease (for ΔC) treatment was performed with re-circularization. The plasmids were verified by Sanger sequencing and are available available upon request.

For in vitro transcription, SmaI-linearized *SMANTIS* constructs were purified by DNA-EtOH precipitation and DNA was subjected to in vitro transcription with the HiScribe T7 High Yield RNA Synthesis Kit (NEB). Afterwards, DNA was digested with RQ DNase I (Promega). The remaining RNA was purified with the RNeasy Mini Kit (Qiagen).

For the in vitro binding assay, 50 ng of in vitro transcribed *SMANTIS* RNA was heated for 2 min at 90 °C in RNA folding buffer (10 mM Tris pH 7.4, 100 mM KCl, 10 mM MgCl_2_), and then put on RT for 20 min to allow RNA folding. The binding reaction of 500 ng recombinant RUNX1 (Origene, TP323854) with 50 ng in vitro transcribed folded *SMANTIS* RNA (and mutants) was performed in buffer R (20 mM Tris/HCl pH 8.0, 150 mM KCl, 2 mM EDTA pH 8.0, 5 mM MgCl_2_, 20 U/ml SUPERaseIn RNase Inhibitor (ThermoFisher Scientific)) for 2 h with a head-over-tail rotator at 4 °C. Afterwards, 2 µl anti-RUNX1/AML1 (ab240639, Abcam) was used for 3 h on the head-over-tail rotator at 4 °C, followed by an incubation with 50 µl DiaMag protein A and protein G coated magnetic beads (Diagenode, Seraing, Belgium) for 1 h at 4 °C. Subsequently, the beads were washed three times for 5 min at 4 °C with buffer R. Elution and purification of the RNAs was done with TRIzol (Thermo Fisher) followed by reverse transcription and RT-qPCR with primers targeting the T7 start region (forward 5’-ACG ACT CAC TAT AGG GAG AC-3’ and reverse 5’-GGT ACC AAG CTT AAG TTT AAA C-3’), which is identical in all *SMANTIS* mutants. For analysis, absolute expression values were normalized to the WT full-length *SMANTIS* sample with the AriaMX qPCR software Version 1.7 (Agilent).

### RUNX1 constructs, in vitro translation and binding assay

pcDNA3.1+RUNX1-6xHis and mutants (ΔRUNT, ΔTAD, ΔC-404, ΔC-178, and ΔN-49) were obtained from Biomatik (Canada) (Supplementary Data 6). The plasmids were verified by sequencing. In vitro protein synthesis of RUNX1-6xHis and its mutants was performed with the PURExpress kit (E6800, NEB) according to the manufacturer’s protocol. The in vitro binding assay of RUNX1-6xHis and its mutants with the in vitro transcribed Alu element region of SMANTIS was done as described above in buffer R with Anti-His tag (sc-8036, Santa Cruz Biotechnology) antibody. After elution and purification of the RNA with TRIzol (Thermo Fisher), reverse transcription and RT-qPCR (AriaMX qPCR software Version 1.7 (Agilent)) was performed with the following primer detecting the Alu element region: forward, 5’-ATC TCC TGA CCT CGT GAT CC-3’ and reverse 5’-GGC TTG GTT GCT TCC GTA TG-3’.

### Overexpression of *SMANTIS*

Overexpression of the Alu element and *SMANTIS* was performed with the plasmids pEGFP-C1-pEF1a-SMTS_Alu (*SMANTIS*-Alu) and pcDNA3.1(+)-SMANTIS (full length), which were obtained from Biomatik (Canada). pEGFP-C1 was obtained from Clonetech (Switzerland) and served as a negative control. For overexpression, transfection of the plasmids was performed by electroporation. 3 × 10^6^ cells were resuspended in 440 µL electroporation E2 buffer. 18 µg of plasmids were electroporated into the cells using the NEON electroporation system (Invitrogen) with four impulses (1400 V and 20 ms per pulse). Cells were cultured in RPMI only with 8% heat-inactivated FCS overnight. At the next day, fresh full growth media was added. Cells were incubated for 24 h and subsequently prepared for adhesion assay by flow cytometry as described above.

### Differentiation of inducible peripheral stem cells into monocytes

The differentiation of inducible peripheral stem cells (iPSC) into monocytes was performed by following the instructions of the STEMdiff Monocyte Kit purchased from STEMCELL Technologies (#05320, STEMdiff^TM^ Monocyte Kit, STEMCELL Technologies). Briefly, iPSC (20 aggregates/cm^2^) were seeded on matrigel coated six well plates. After adhesion of the cells, medium A was added and cells were incubated for 2 days (mesodermal phase). Medium was changed at the third day to medium B to generate hematopoietic progenitor cells. During the following three days, media was diluted with fresh media by removing used and adding fresh media. On day 7, monocyte differentiation was initiated by changing media to monocyte differentiation medium and incubated for 3 d. Differentiated monocytes could be harvested from the supernatant from day 14 on. Monocytes were harvested at day 15 and further differentiated macrophage-like cells were harvested after 21 d. iPSC (d0), monocytes (d15), and further differentiated monocytes (d21) were used for RNA- and ATAC-seq as described above.

### Statistics and Reproducibility

Unless otherwise indicated, data are shown as means ± SD. Statistical calculation was performed with GraphPad Prism 10.1.2. For multiple group comparisons, ANOVA followed by post hoc testing was performed. For multiple group comparison with unequal variance Brown-Forsythe and Welch ANOVA with Games-Howell post hoc was applied. Statistics of two groups was either performed with unpaired or paired t-test or if no normal distribution was assumed with Mann-Whitney t-test. Significance was considered if the *p* values were <0.05. Unless otherwise indicated, n represents the number of individual performed experiments. Except of the iPSC and osteoclast-differentiation RNA-seq experiments, which were performed *n* = 2, all other experiments were performed at least *n* = 3 as biological replicates. For experiments involving cultured cells, all replicates were performed with cells from the same passage. Numerical source data of the graphs presented in the main manuscript are uploaded as Supplementary Data 12.

### Reporting Summary

Further information on research design is available in the Nature Portfolio Reporting Summary linked to this article.

## Supplementary information

Supplementary Information

Description of Additional Supplementary File

Supplementary Data 1

Supplementary Data 2

Supplementary Data 3

Supplementary Data 4

Supplementary Data 5

Supplementary Data 6

Supplementary Data 7

Supplementary Data 8

Supplementary Data 9

Supplementary Data 10

Supplementary Data 11

Supplementary Data 12

Reporting Summary

## Data Availability

The mass spectrometry proteomics data have been deposited to the ProteomeXchange Consortium via the PRIDE^64^ partner repository with the dataset identifier PXD048847. Raw genomic data of the AML patient cohort^16^ was retrieved from the European Genome-Phenome Archive (EGA) with the study accession ID EGA: EGAS00001005950 and Whole transcriptome RNA sequencing data EGA: EGAD00001008484. The ATAC-Seq datasets have been deposited and are available at NCBI GEO with the accession number GSE254673: https://www.ncbi.nlm.nih.gov/geo/query/acc.cgi?acc=GSE254673 The CUT&RUN dataset have been deposited and are available at NCBI GEO with the accession number GSE254674: https://www.ncbi.nlm.nih.gov/geo/query/acc.cgi?acc=GSE254674. The RNA-Seq datasets of NTC, knockout of *SMANTIS*, knockout of RUNX1 (all untreated) have been deposited and are available at NCBI GEO with the accession number GSE254679: https://www.ncbi.nlm.nih.gov/geo/query/acc.cgi?acc=GSE254679. The RNA-Seq datasets after differentiation of inducible pluripotent stem cells into monocytes or further into macrophages have been deposited and are available at NCBI GEO with the accession number GSE254680: https://www.ncbi.nlm.nih.gov/geo/query/acc.cgi?acc=GSE254680. The RNA-Seq datasets, where NTC, knockout of *SMANTIS*, knockout of RUNX1 THP-1 were differentiated into osteoclast-like cells, have been deposited and are available at NCBI GEO with the accession number GSE254681: https://www.ncbi.nlm.nih.gov/geo/query/acc.cgi?acc=GSE254681.
