# Supplementary material for: RUNX1 interacts with lncRNA SMANTIS to regulate monocytic cell functions
Source: Commun Biol. 2024 Sep 13;7:1131. doi: 10.1038/s42003-024-06794-2 (PMC11399395; doi:10.1038/s42003-024-06794-2)
Supplement: Supplementary file 1 — Supplementary Information [file 42003_2024_6794_MOESM1_ESM.pdf]

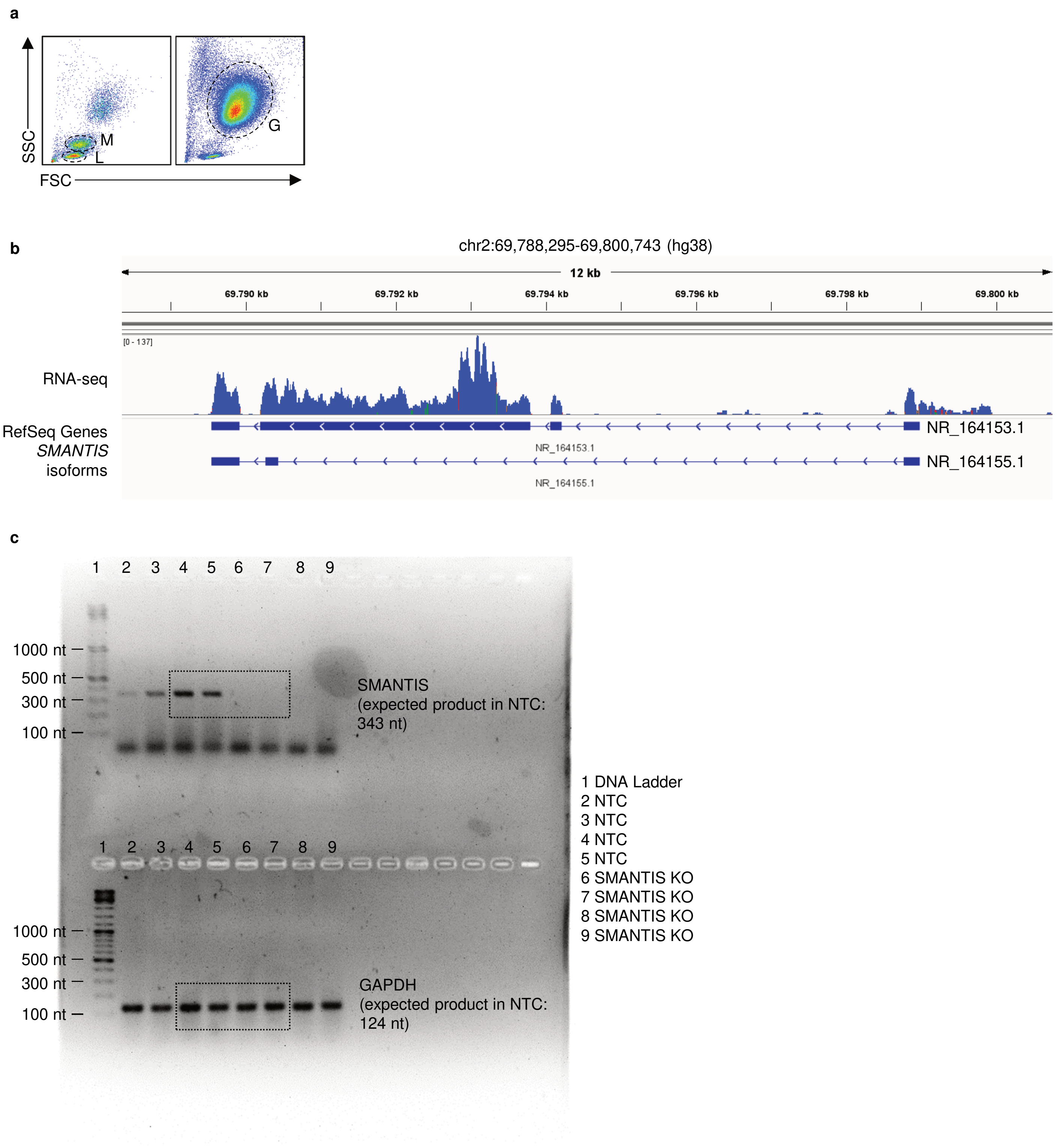

Supplemental figure 1

a Gating strategy for the FACS isolated human blood cells (granulocytes (G), monocytes (M), lymphocytes (L)). **b** IGV browser trace of RNA-seq in monocytes differentiated from iPSCs. The locus of both *SMANTIS* isoforms (NR_164153.1 (long isoform) and NR_164155.1 (short isoform)) is shown (chr2:69,789,492-69,789,645 (hg38)). **c** Uncropped image of Fig. 2b. Agarose gel electrophoresis after PCR of the genomic DNA region of SMANTIS. NTC, non-targeting control gRNA. GAPDH serves as loading control. The dashed box shows the cropped image.

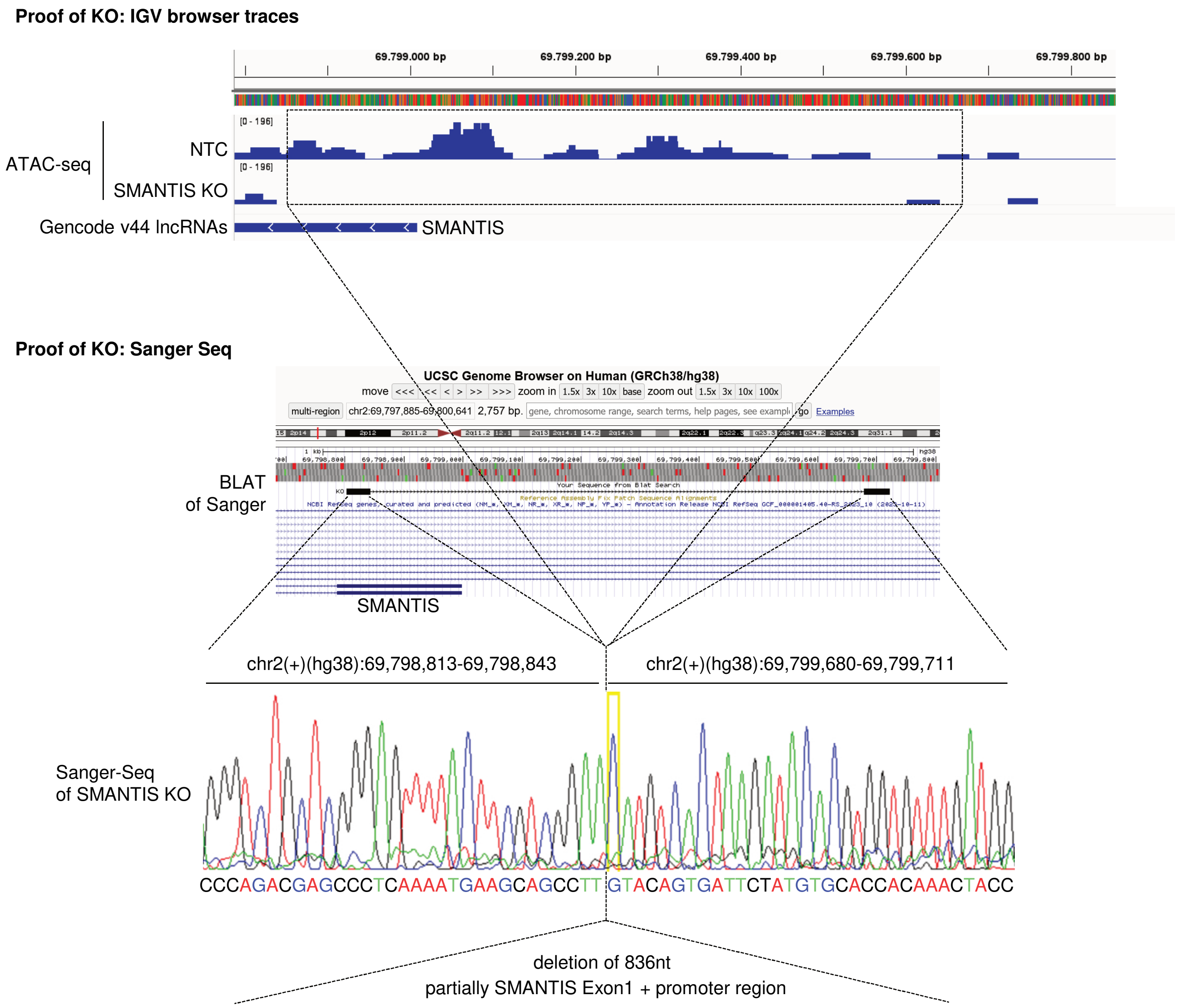

>deleted_region_hg38_dna range=chr2:69,798,844-69,799,679
5‘_GTTGTCTGGGGTGAATACTGAGGTTCTTGGTCACACGACCAGATATTGAGGTCAAGGACATGCACACACACATC
GAGTGAGTTTGGAGCAGGAGTTTAATAGGCAAAAGAACAGAACAGCTCACAGTCACAGAGAGGGGTCCCGAGCAG
GTTGCTAAGCAGTAGTAAAAATGTCAGGGTTTTTATAAATGGTCTAGGGAGGAGGGGCCTCATGAGGAGGGGATG
TCTTATCCTCTTAGGGCCTGATGGTTTAGCTGCGACCAAGTGTGCTATCTATATAGAGCAGAATTTTTTTTATCAGCT
TTTATCCCATTCCCTGACCACATAGGCTGACTCTTAGTCTGTGTTTCTTTGTCTTGCTTATCTGGGAGGGAGAGTTT
CTGTGTCTGTTCCCATACATCTTCTTGCAGCTGCAGGCATCCGCCCCATGAGTCTGCTTTTAGTTTCTCTATCTTAG
TGCACCTAAAGGGAAAGGAATGTGCTTATTAAGACCCACTGTTTTACTGGGGCCCATTGTATAAGTGTGAAGTTTG
GCAATTACCCAGGAGACTTCCACCCCTTCTTCTGTGCTTGAGCTGTCTTATCTGTATTTTACAGCCTGAACTTTCAG
GTTACTTGTTGTTAGAAGAGAAGTGATTTTTTTTAACTGTGTGTGGTTAGAAAGGGAGCTATTTTTGAGCTGCTTTTT
GTTAAAAGGAAAGTTTTCTGCTGGGGACTCACTTTACCCTAAATGTCTACCTAAATAATTTCTTTTTGCCTCCTATAA
CACTGGTAGTGTCTTCCAGCATGCTACCCTCCAGTTAGCTCCTTGGGGGCCAACACCTTCATGCTGGGTAG_3‘

Supplemental figure 2

Genomic locus, LentiCRISPR strategy and sanger sequencing for the selected knockout clone of *SMANTIS*. A dual gRNA approach was used to target the transcriptional start site and parts of the promoter of *SMANTIS*. LentiCRISPRv2 targeting *SMANTIS* led to a deletion of 836 nucleotides, which covered partially *SMANTIS* Exon1, the transcriptional start site and parts of the promoter region.

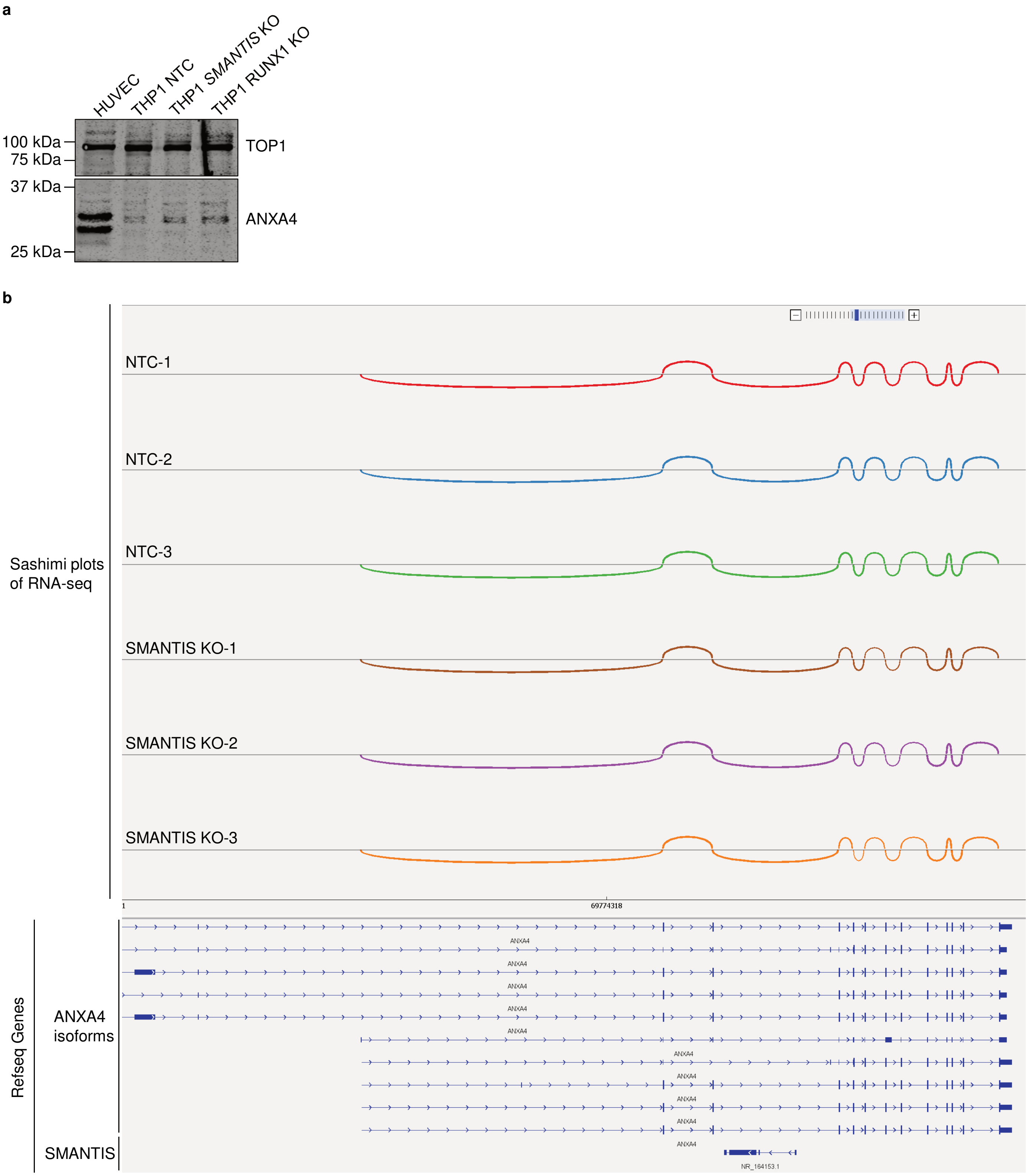

Supplemental figure 3
a Western blot analysis with antibodies against ANXA4 in HUVEC and THP-1 cells (NTC, *SMANTIS* KO and RUNX1 KO). Topoisomerase I (TOP1) served as loading control. **b** Sashimi plots of RNA-seq data after *SMANTIS* knockout. The *ANXA4* locus is shown.

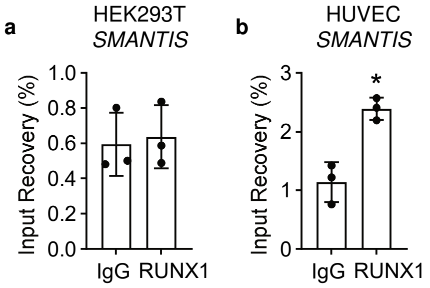

Supplemental figure 4
a&b RNA-immunoprecipitation (RIP) in HEK293T (a) or human umbilical vein endothelial cells (HUVEC) (b) with antibodies against RUNX1 or IgG control followed by RT-qPCR of *SMANTIS*. HEK293T, n=3; HUVEC, n=3; paired t-test. Error bars are mean +/- SD. *p<0.05. KO, knockout.

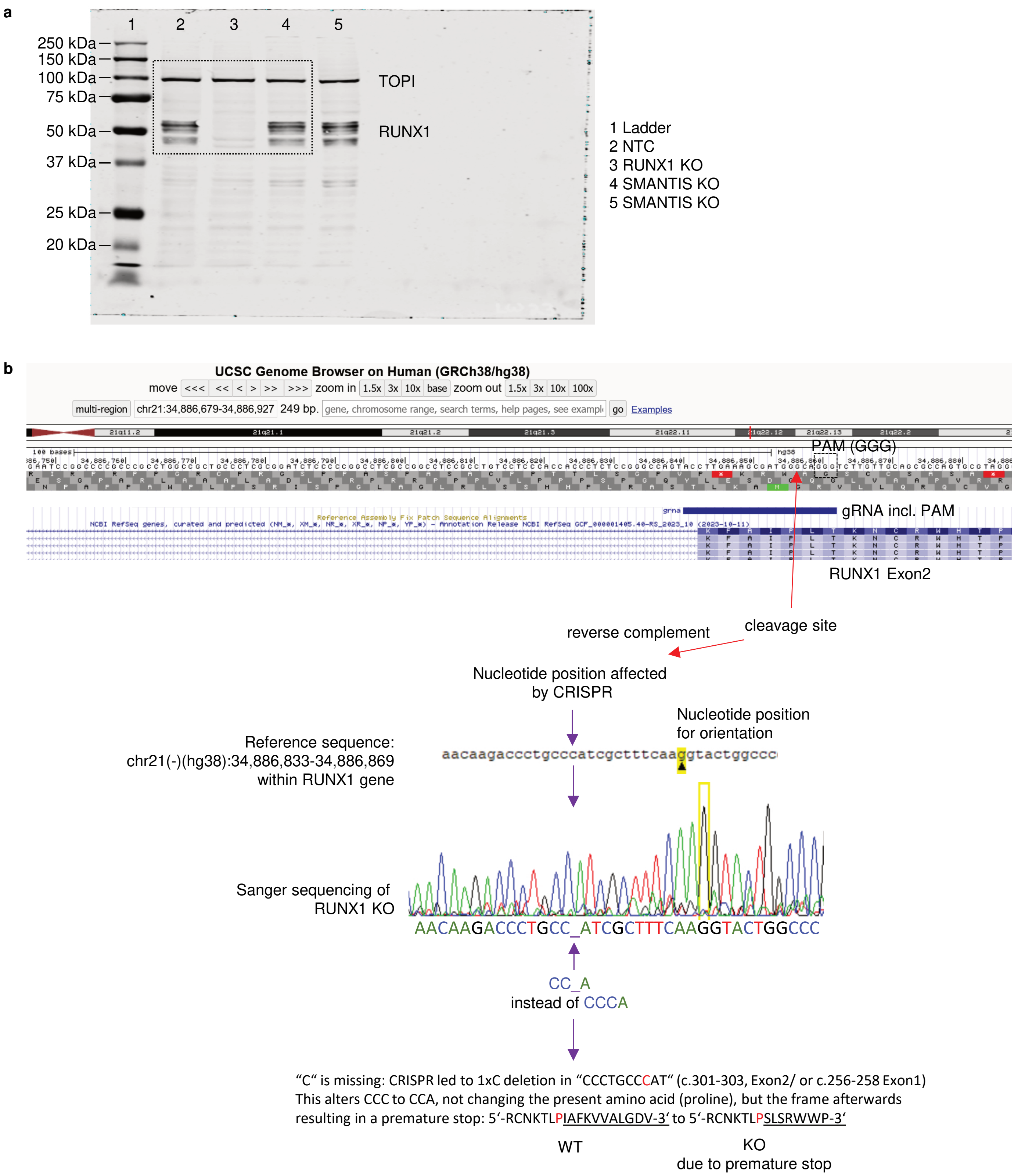

Supplemental figure 5

a Uncropped image of Fig. 3b. Western blot analysis with antibodies against RUNX1 and topoisomerase I (TOP1) of control (NTC), RUNX1 knockout and *SMANTIS* KO in THP-1 cells. The dashed box shows the cropped image. **b** Genomic locus, LentiCRISPR strategy and sanger sequencing for the selected knockout clone of *RUNX1*. A single gRNA approach was used to target exon2 of *RUNX1*. LentiCRISPRv2 with a gRNA targeting *RUNX1* exon2 led to a deletion of one "C" within "CCCA", which altered the sequence to "CCA", changing the reading frame resulting in a premature stop.

a

Total Target Sequences = 657, Total Background Sequences = 2675

Rank	Motif	Name	P-value	log P-pvalue	q-value (Benjamini)	# Target Sequences with Motif	% of Targets Sequences with Motif	# Background Sequences with Motif	% of Background Sequences with Motif	Motif File	SVG
1	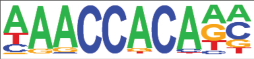	RUNX1(Runt)/Jurkat-RUNX1-ChIP-Seq(GSE29180)/Homer	1e-212	-4.899e+02	0.0000	425.0	64.69%	185.4	6.93%	motif file (matrix)	svg
2	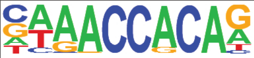	RUNX(Runt)/HPC7-Runx1-ChIP-Seq(GSE22178)/Homer	1e-201	-4.645e+02	0.0000	391.0	59.51%	146.9	5.49%	motif file (matrix)	svg
3	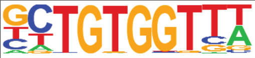	RUNX-AML(Runt)/CD4+-PolII-ChIP-Seq(Barski_et_al.)/Homer	1e-187	-4.320e+02	0.0000	346.0	52.66%	101.8	3.80%	motif file (matrix)	svg
4	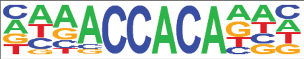	RUNX2(Runt)/PCa-RUNX2-ChIP-Seq(GSE33889)/Homer	1e-178	-4.114e+02	0.0000	379.0	57.69%	172.3	6.43%	motif file (matrix)	svg
5	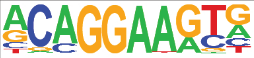	ERG(ETS)/VCaP-ERG-ChIP-Seq(GSE14097)/Homer	1e-46	-1.062e+02	0.0000	172.0	26.18%	149.9	5.60%	motif file (matrix)	svg

b

Total Target Sequences = 807, Total Background Sequences = 3308

Rank	Motif	Name	P-value	log P-pvalue	q-value (Benjamini)	# Target Sequences with Motif	% of Targets Sequences with Motif	# Background Sequences with Motif	% of Background Sequences with Motif	Motif File	SVG
1	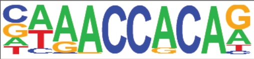	RUNX(Runt)/HPC7-Runx1-ChIP-Seq(GSE22178)/Homer	1e-96	-2.221e+02	0.0000	254.0	31.47%	137.6	4.16%	motif file (matrix)	svg
2	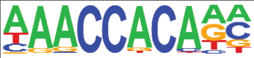	RUNX1(Runt)/Jurkat-RUNX1-ChIP-Seq(GSE29180)/Homer	1e-92	-2.120e+02	0.0000	273.0	33.83%	183.6	5.55%	motif file (matrix)	svg
3	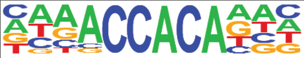	RUNX2(Runt)/PCa-RUNX2-ChIP-Seq(GSE33889)/Homer	1e-89	-2.053e+02	0.0000	259.0	32.09%	166.6	5.03%	motif file (matrix)	svg
4	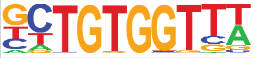	RUNX-AML(Runt)/CD4+-PolII-ChIP-Seq(Barski_et_al.)/Homer	1e-82	-1.900e+02	0.0000	224.0	27.76%	124.3	3.76%	motif file (matrix)	svg
5	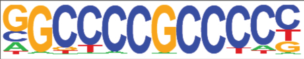	Sp1(Zf)/Promoter/Homer	1e-31	-7.268e+01	0.0000	95.0	11.77%	57.9	1.75%	motif file (matrix)	svg

c

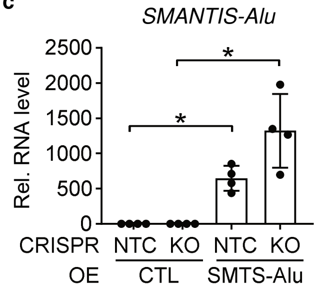

Supplemental figure 6

a-b Motif enrichment analysis with HOMER (v4.11) of the RUNX1 CUT&RUN data after *SMANTIS* knockout. Briefly, unchanged (a, common) or downregulated (b) RUNX1 CUT&RUN peak sequences were extracted from the GRCh38 genome assembly using bedtools (v2.27.1) getfasta (cite PMID: 20110278). These sequences served as input to findMotifs.pl from HOMER. The top 5 predicted motifs are shown. **c** RT-qPCR of *SMANTIS-Alu* after overexpression of the *SMANTIS-Alu* element or an empty control vector (CTL) in NTC or *SMANTIS* KO THP-1 cells. NTC overexpressing CTL were set to 1. One-Way ANOVA with Tukey's post-hoc test. n=4. Error bars are mean +/- SD. *p<0.05. KO, knockout.

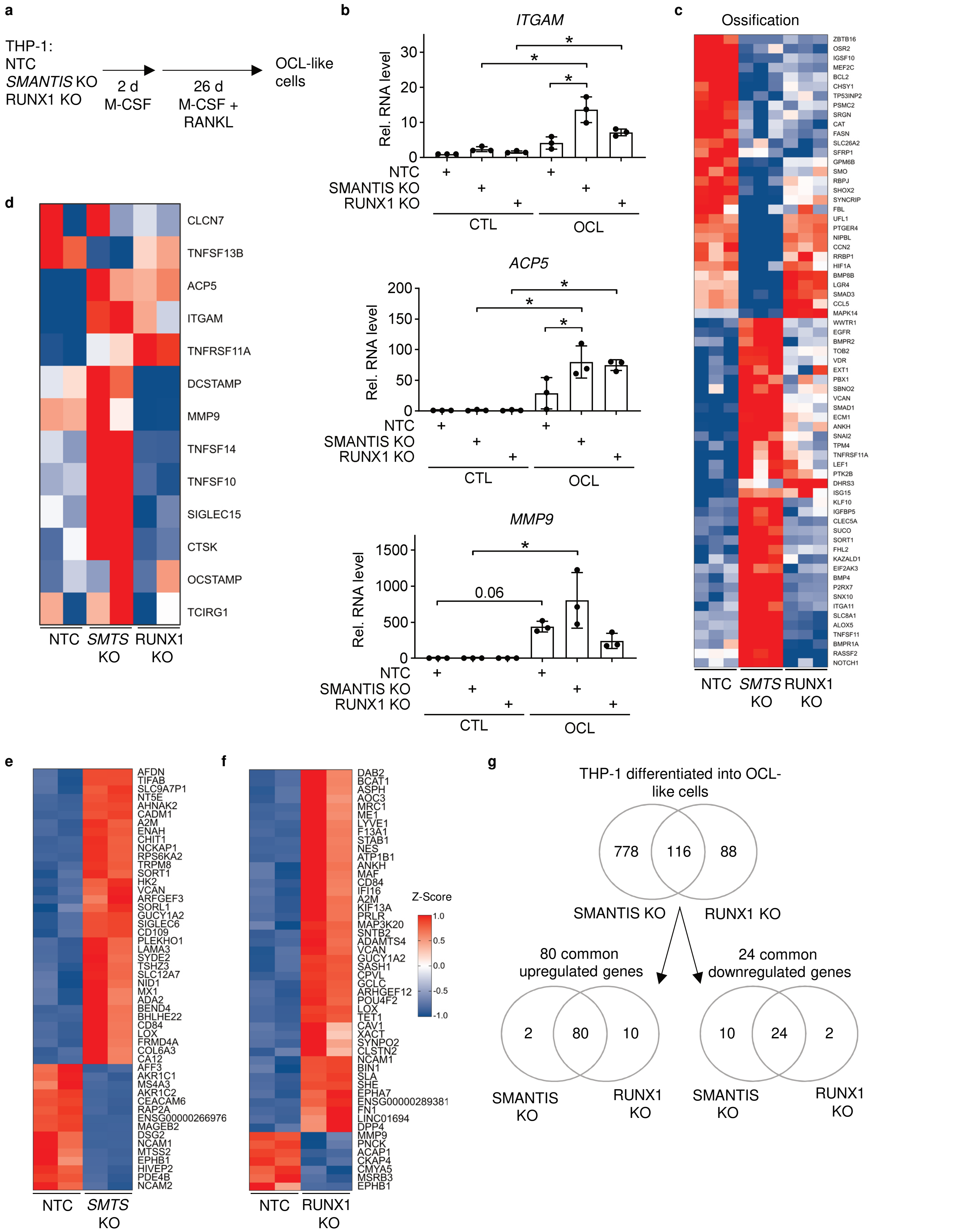

Supplemental figure 7

a Schematic representation of the differentiation of THP-1 cells into osteoclast-like cells (OCL-like cells). M-CSF, Macrophage colony-stimulating factor; RANKL, receptor activator of nuclear factor-κB-ligand. **b** RT-qPCR of osteoclast marker ITGAM, ACP5 and MMP9 after treatment of NTC, *SMANTIS* KO, and RUNX1 KO for four weeks with osteoclast medium (OCL) or without treatment (CTL). n=3, Ordinary one-way ANOVA with Tukey post hoc test. **c** Heat map of the differentially regulated genes associated with ossification determined by RNA-Seq. n=3. *SMANTIS*, SMTS. **d** Osteoclast marker expressed in NTC, *SMANTIS* KO, and RUNX1 KO after osteoclast differentiation determined with RNA-Seq. n=2. *SMANTIS*, SMTS. **e, f** TOP 50 differentially expressed genes using Z-score in *SMANTIS* KO (e) and RUNX1 KO (f) after differentiation into OCL-like cells deterimined with RNA-Seq. n=2. *SMANTIS*, SMTS. **g** Venn diagrams of the overlapping differentially expressed genes of *SMANTIS* KO and RUNX1 KO. Error bars are mean +/- SD. *p<0.05. KO, knockout.
