# Supplementary material for: RUNX1 interacts with lncRNA SMANTIS to regulate monocytic cell functions
Source: Commun Biol. 2024 Sep 13;7:1131. doi: 10.1038/s42003-024-06794-2 (PMC11399395; doi:10.1038/s42003-024-06794-2)
Supplement: Supplementary file 2 — Description of Additional Supplementary File [file 42003_2024_6794_MOESM2_ESM.pdf]

Description of Additional Supplementary Files

File name: Supplementary Data 1
Description: Differentially expressed genes from iPSC-monocyte-macrophage differentiation (day 15 versus day 0)

File name: Supplementary Data 2
Description: Differentially expressed genes from iPSC-monocyte-macrophage differentiation (day 21 versus day 0)

File name: Supplementary Data 3
Description: Differentially expressed genes from iPSC-monocyte-macrophage differentiation (day 21 versus day 15)

File name: Supplementary Data 4
Description: Individual AML patient expression values

File name: Supplementary Data 5
Description: Proteins identified after antisense oligonucleotide pulldown with SMANTIS probes in NTC vs. SMANTIS KO THP-1 File name:

Supplementary Data 6
Description: RUNX1 mutant sequences

File name: Supplementary Data 7
Description: Differentially expressed genes after RNA-Seq of SMANTIS knockout versus NTC.

File name: Supplementary Data 8
Description: Differentially expressed genes after RNA-Seq of RUNX1 knockout versus NTC

File name: Supplementary Data 9
Description: CUT&RUN with anti-RUNX1 in SMANTIS knockout cells

File name: Supplementary Data 10
Description: Differentially expressed genes after OCL-differentiation RNA-Seq of SMANTIS knockout versus NTC

File name: Supplementary Data 11

Description: Differentially expressed genes after OCL-differentiation RNA-Seq of RUNX1 knockout versus NTC

File name: Supplementary Data 12
Description: Source Data
