# Supplementary material for: RUNX1 interacts with lncRNA SMANTIS to regulate monocytic cell functions
Source: Commun Biol. 2024 Sep 13;7:1131. doi: 10.1038/s42003-024-06794-2 (PMC11399395; doi:10.1038/s42003-024-06794-2)
Supplement: Supplementary file 15 — Reporting Summary [file 42003_2024_6794_MOESM15_ESM.pdf]

nature portfolio

Corresponding author(s): Matthias S. Leisegang

Last updated by author(s): Aug 13, 2024

Reporting Summary

Nature Portfolio wishes to improve the reproducibility of the work that we publish. This form provides structure for consistency and transparency in reporting. For further information on Nature Portfolio policies, see our Editorial Policies and the Editorial Policy Checklist.

Statistics

For all statistical analyses, confirm that the following items are present in the figure legend, table legend, main text, or Methods section.

n/a	Confirmed	
☐	☒	The exact sample size (*n*) for each experimental group/condition, given as a discrete number and unit of measurement
☐	☒	A statement on whether measurements were taken from distinct samples or whether the same sample was measured repeatedly
☐	☒	The statistical test(s) used AND whether they are one- or two-sided<br>*Only common tests should be described solely by name; describe more complex techniques in the Methods section.*
☒	☐	A description of all covariates tested
☐	☒	A description of any assumptions or corrections, such as tests of normality and adjustment for multiple comparisons
☐	☒	A full description of the statistical parameters including central tendency (e.g. means) or other basic estimates (e.g. regression coefficient) AND variation (e.g. standard deviation) or associated estimates of uncertainty (e.g. confidence intervals)
☐	☒	For null hypothesis testing, the test statistic (e.g. *F*, *t*, *r*) with confidence intervals, effect sizes, degrees of freedom and *P* value noted<br>*Give P values as exact values whenever suitable.*
☒	☐	For Bayesian analysis, information on the choice of priors and Markov chain Monte Carlo settings
☒	☐	For hierarchical and complex designs, identification of the appropriate level for tests and full reporting of outcomes
☒	☐	Estimates of effect sizes (e.g. Cohen's *d*, Pearson's *r*), indicating how they were calculated

Our web collection on statistics for biologists contains articles on many of the points above.

Software and code

Policy information about availability of computer code

Data collection	Raw genomic data of the AML patient cohort was retrieved from the European Genome-Phenome Archive (EGA) with the study accession ID EGA: EGAS00001005950 and Whole transcriptome RNA sequencing data EGA: EGAD00001008484. published at Jayavelu, A. K. et al. The proteogenomic subtypes of acute myeloid leukemia. Cancer cell 40, 301-317.e12; 10.1016/j.ccell.2022.02.006 (2022)
Data analysis	Cell Sorter Software Version 2.1.6 (Sony Biotechnology), FlowJo version 10.7.1, AriaMX qPCR software Version 1.7 (Agilent), Bowtie2 (v2.4.5), samtools rmdup (v1.1.0), bamCoverage, MACS3 (v3.0.0), Genomics Viewer (IGV) (Version 1.16.2), Trimmomatic version 0.39, human genome version hg38 (Ensembl release 104 or 109) with STAR 2.7.10a, Picard 2.25.5, or Picard 3.0.0, featureCounts 2.0.2, or featureCounts 2.0.4, DESeq2 version 1.30.1 or version 1.36.0, hg38 genome and quantified using Salmon (v1.5.2), RStudio (R version 4.1.2), samtools fixmate and samtools markdup –r, respectively (v1.1.0), bedtools intersect –v (v2.27.1), HOMER annotatePeaks.pl (v4.11), bedGraphToBigWig from the kentUtils collection of tools from UCSC, ZEN 3.2 (blue edition) software (Carl Zeiss Microscopy GmbH), mage J-win.64 v1.54h (BioVoxxel, Germany), Image Studio Version 5.2.5 (Licor), the fluorescence microscope BZ-X800 (Version 01.03.00.001, Keyence, Germany), BZ-X800 Analyzer (Version 1.1.2.4, Keyence, Germany), MaxQuant 2.0.1.0, Perseus 1.6.1.3, Excel (Microsoft Office 2016), human reference proteome set (Uniprot, August 2023, 104436 entries), GraphPad Prism 10.1.2

For manuscripts utilizing custom algorithms or software that are central to the research but not yet described in published literature, software must be made available to editors and reviewers. We strongly encourage code deposition in a community repository (e.g. GitHub). See the Nature Portfolio guidelines for submitting code & software for further information.

Data

Policy information about availability of data

All manuscripts must include a data availability statement. This statement should provide the following information, where applicable:

- Accession codes, unique identifiers, or web links for publicly available datasets
- A description of any restrictions on data availability
- For clinical datasets or third party data, please ensure that the statement adheres to our policy

Data availability
The mass spectrometry proteomics data have been deposited to the ProteomeXchange Consortium via the PRIDE64 partner repository with the dataset identifier PXD048847. Please use the following login details: Username: reviewer_pxd048847@ebi.ac.uk and Password: HbcqN5dC.
Raw genomic data of the AML patient cohort16 was retrieved from the European Genome-Phenome Archive (EGA) with the study accession ID EGA: EGAS00001005950 and Whole transcriptome RNA sequencing data EGA: EGAD00001008484.
The ATAC-Seq datasets have been deposited and are available at NCBI GEO with the accession number GSE254673: https://www.ncbi.nlm.nih.gov/geo/query/acc.cgi?acc=GSE254673

The CUT&RUN dataset have been deposited and are available at NCBI GEO with the accession number GSE254674: https://www.ncbi.nlm.nih.gov/geo/query/acc.cgi?acc=GSE254674

The RNA-Seq datasets of NTC, knockout of SMANTIS, knockout of RUNX1 (all untreated) have been deposited and are available at NCBI GEO with the accession number GSE254679: https://www.ncbi.nlm.nih.gov/geo/query/acc.cgi?acc=GSE254679

The RNA-Seq datasets after differentiation of inducible pluripotent stem cells into monocytes or further into macrophages have been deposited and are available at NCBI GEO with the accession number GSE254680: https://www.ncbi.nlm.nih.gov/geo/query/acc.cgi?acc=GSE254680

The RNA-Seq datasets, where NTC, knockout of SMANTIS, knockout of RUNX1 THP-1 were differentiated into osteoclast-like cells, have been deposited and are available at NCBI GEO with the accession number GSE254681: https://www.ncbi.nlm.nih.gov/geo/query/acc.cgi?acc=GSE254681

Research involving human participants, their data, or biological material

Policy information about studies with human participants or human data. See also policy information about sex, gender (identity/presentation), and sexual orientation and race, ethnicity and racism.

Reporting on sex and gender	Sex and gender were not considered and evaluated in this study
Reporting on race, ethnicity, or other socially relevant groupings	Race, ethnicity, or other socially relevant groupings were not considered and evaluated in this study
Population characteristics	Population characteristics of the human research participants were not evaluated in this study.
Recruitment	This study did not in involve active recruitment of participants.<br>Human peripheral blood mononuclear cells (PBMC) and granulocytes were isolated from commercially available buffy coats of anonymous donors (DRK Blutspendedienst Baden-Württemberg-Hessen, Institut für Transfusionsmedizin und Immunhämatologie, Frankfurt, Germany).
Ethics oversight	Raw sequencing data of the acute myeloid leukemia (AML) patient cohort were obtained from following resource as well as all relevant informations on AML patient cohort are listed here: European Genome-Phenome Archive (EGA) with the study accession ID EGA: EGAS00001005950 and Whole transcriptome RNA sequencing data EGA: EGAD00001008484 and published at Jayavelu, A. K. et al. The proteogenomic subtypes of acute myeloid leukemia. Cancer cell 40, 301-317.e12; 10.1016/j.ccell.2022.02.006 (2022)

Note that full information on the approval of the study protocol must also be provided in the manuscript.

Field-specific reporting

Please select the one below that is the best fit for your research. If you are not sure, read the appropriate sections before making your selection.

☒ Life sciences ☐ Behavioural & social sciences ☐ Ecological, evolutionary & environmental sciences

For a reference copy of the document with all sections, see nature.com/documents/nr-reporting-summary-flat.pdf

Life sciences study design

All studies must disclose on these points even when the disclosure is negative.

Sample size	Sample size were chosen based on standards and previous experiences in the field.
Data exclusions	No data was excluded.
Replication	Except of the iPSC and osteoclast-differentiation RNA-seq experiments, which were performed n=2, all other experiments were performed at least n=3 as biological replicates.
Randomization	not used
Blinding	not used

Reporting for specific materials, systems and methods

We require information from authors about some types of materials, experimental systems and methods used in many studies. Here, indicate whether each material, system or method listed is relevant to your study. If you are not sure if a list item applies to your research, read the appropriate section before selecting a response.

Materials & experimental systems

n/a	Involved in the study	
☐	☒	Antibodies
☐	☒	Eukaryotic cell lines
☒	☐	Palaeontology and archaeology
☒	☐	Animals and other organisms
☒	☐	Clinical data
☒	☐	Dual use research of concern
☒	☐	Plants

Methods

n/a	Involved in the study	
☒	☐	ChIP-seq
☐	☒	Flow cytometry
☒	☐	MRI-based neuroimaging

Antibodies

Antibodies used	Recombinant Anti-RUNX1/AML1 (ab240639, Abcam), Anti-RUNX1 (AF2399, R&D Systems, for PLA), Anti-TopI (sc5342, Santa Cruz), Anti-EP300 (A300358A, Bethyl), Anti-SIN3A (C15410250, Diagenode), Anti-CBFB (ab125191, Abcam), Anti-ANXA4 (sc-46693, Santa Cruz) and Anti-His tag (sc-8036, Santa Cruz Biotechnology).
Validation	All antibodies are commercially available and validated by the companies.

Eukaryotic cell lines

Policy information about cell lines and Sex and Gender in Research

Cell line source(s)	Human monocytic cell line THP-1 (from ATCC, LGC Promochem, Wesel, Germany, cat. No. TIB-202)<br>Pooled human umbilical vein endothelial cells (HUVEC) purchased from PromoCell (C12203, Lot number 474Z010, 471Z011, 466Z022, Heidelberg, Germany)<br>Human embryonic kidney 293 cells (HEK293) were purchased from ATCC (Manassas, USA)<br>Lenti-X293T cells from Takara (632180, Japan)
Authentication	None of the cell line were authenticated.<br>iPSC were checked for iPSC marker genes. HUVEC and THP-1 were checked with RNA-Seq.<br>HEK293 and Lenti-X293T cells served as protein purification and virus production purposes.
Mycoplasma contamination	All cell lines were regularly tested negative for Mycoplasma contamination.
Commonly misidentified lines<br>(See ICLAC register)	No commonly misidentified lines were used

Plants

Seed stocks: not used

Novel plant genotypes: not used

Authentication: not used

Flow Cytometry

Plots

Confirm that:

- [ ] The axis labels state the marker and fluorochrome used (e.g. CD4-FITC).
- [ ] The axis scales are clearly visible. Include numbers along axes only for bottom left plot of group (a 'group' is an analysis of identical markers).
- [ ] All plots are contour plots with outliers or pseudocolor plots.
- [ ] A numerical value for number of cells or percentage (with statistics) is provided.

Methodology

Sample preparation: Isolated blood cells and cells of the adhesion assay were resuspened in FACS Buffer without any fluorescent antibodies. Cells from the adhesion assay were stained prior with Vybrant DiI Cell-Labeling Solution (V-22885, Thermo Fisher Scientific).

Instrument: SH800S Cell Sorter from Sony Biotechnology

Software: Cell Sorter Software Version 2.1.6 (Sony Biotechnology)

Cell population abundance: For cells isolated from blood: relevant cells were purified by removing red blood cells. Subsequently, cells were separated with Ficoll, which is a synthetic branched saccharide-epichlorhydrin copolymer (density of 1.077 g/mL). Based on the cells density PBMC could be separated from granulocytes. PBMC were further separated by their size and granularity with flow cytometry.

Gating strategy: Isolated blood cells were analysed by size indicated by forward scatter (FSC) and granularity indicated by sideward scatter (SSC). For the adhesion assay, negative and positive controls for Vybrant DiI Cell-Labeling Solution were checked and positive cell population was adjusted respectivley.

- [x] Tick this box to confirm that a figure exemplifying the gating strategy is provided in the Supplementary Information.
